# Enhancement of Cisplatin Efficacy by the Histone Demethylase Inhibitor JIB-04 in Head and Neck Squamous Cell Carcinoma (HNSCC) Cell Lines

**DOI:** 10.3390/cells15212023

**Published:** 2026-11-06

**Authors:** Kamila Adamczuk, Grzegorz Adamczuk, Michał Kiełbus, Matthias Nees, Andrzej Stepulak

**Affiliations:** 1 Department of Biochemistry and Molecular Biology, Medical University of Lublin, 20-093 Lublin, Poland; michal.kielbus@umlub.edu.pl (M.K.); matthias.nees@umlub.edu.pl (M.N.); 2 Department of Physiology and Toxicology, The John Paul II Catholic University of Lublin, 20-708 Lublin, Poland; grzegorz.adamczuk@kul.pl

**Keywords:** head and neck squamous cell carcinoma, HNSCC, cisplatin, JIB-04, histone demethylase inhibitor, combination treatment

## Abstract

**Highlights:**

**What are the main findings?**

- A localized positive JIB-04–cisplatin interaction was identified in UT-SCC-24A but was not reproduced consistently across the other established HNSCC cell lines tested.
- Combined treatment reduced DNA synthesis and increased S/G2–M accumulation and apoptosis; RNA-seq revealed stress-response activation and suppression of proliferation-related genes following JIB-04 treatment.

**What are the implications of the main findings?**

- JIB-04 may increase cisplatin sensitivity by limiting the ability of selected HNSCC cells to adapt to treatment-induced stress and resume proliferation.
- The variable response across cell lines suggests that further work is needed to identify which tumors could benefit from this combination.

**Abstract:**

Head and neck squamous cell carcinoma (HNSCC) develops resistance to cisplatin. We investigated whether JIB-04, an inhibitor of Jumonji histone demethylases, enhances cisplatin activity in established HNSCC cell lines. Four UT-SCC cell lines originating from primary tumors and matched lymph-node metastases were treated with JIB-04 and cisplatin, alone or combined. Viability was assessed by MTT assay, and drug interactions were evaluated using the Highest Single Agent model. In UT-SCC-24A cells, we assessed BrdU incorporation, LDH release, cell-cycle distribution, and apoptosis. RNA sequencing was performed after 24 h at the same drug concentrations used in functional assays. JIB-04 reduced viability in all HNSCC lines at submicromolar concentrations, with moderate effects in BJ fibroblasts. HSA analysis identified a localized positive interaction in UT-SCC-24A, where the overall HSA score was 4.25, whereas local scores reached approximately 16–18 within a narrow low-dose range. Co-treatment further reduced DNA synthesis and increased S/G2-M accumulation and apoptosis, without significantly increasing LDH release. RNA sequencing revealed JIB-04-associated induction of integrated stress response/ATF4- and DNA damage response-related genes, alongside suppression of proliferation- and cell-cycle-associated genes. The combination retained these transcriptional features. These findings support the further investigation of JIB-04–cisplatin combinations in selected HNSCC cell-line models.

## 1. Introduction

Cisplatin remains an important component of treatment for head and neck squamous cell carcinoma (HNSCC), but resistance limits its effectiveness. Cisplatin-induced DNA lesions interfere with replication and activate DNA damage responses, cell-cycle checkpoints, and pathways that may ultimately lead to cell death. The response to cisplatin is heterogeneous and may be influenced by reduced intracellular drug accumulation, increased tolerance or repair of DNA damage, and activation of pro-survival programs. Altered drug transport is one such mechanism: in HNSCC cell models, exposure to secondhand smoke extract was associated with reduced expression of the cisplatin influx transporter CTR1 and increased expression of ABCG2 and ATP7A, alongside a reduced response to cisplatin [1]. Together, these observations illustrate the complexity of cisplatin resistance and support the investigation of compounds that may modify treatment response.

Epigenetic regulators are of interest in this context because they participate in the control of transcriptional programs involved in proliferation, DNA damage responses, stress adaptation, and cell survival [2,3,4,5,6,7]. Histone lysine methylation is regulated by lysine methyltransferases and histone lysine demethylases (KDMs) [4,5,6]. Several KDM family members have been associated with HNSCC biology and have been investigated as pharmacological targets. However, the role of individual enzymes in the response to cisplatin remains incompletely defined, and effects observed after treatment with a KDM-directed compound cannot be attributed to a specific demethylase without direct evidence of target engagement.

JIB-04 is a small molecule reported to inhibit several Jumonji C domain-containing histone demethylases, including members of the KDM4 and KDM5 families [8]. A compound with activity across multiple KDMs offers a way to explore cellular responses that might not be captured by inhibiting one enzyme alone; at the same time, this broader pharmacological profile makes it essential to distinguish the effects of JIB-04 treatment from a demonstrated KDM-dependent mechanism.

Studies in non-HNSCC models provide a further rationale for evaluating JIB-04 alongside cisplatin. JIB-04 has been associated with impaired proliferation and cell-cycle progression in hepatocellular carcinoma cells, and with deregulation of oncogenic transcriptional programs and increased DNA damage in Ewing sarcoma cells. In MECOM-amplified ovarian cancer models, JIB-04 was also reported to improve cisplatin response. These findings suggest that JIB-04 can affect several processes relevant to the response to cytotoxic treatment, while emphasizing that the underlying pathways and magnitude of the effect may depend on tumor context [9,10,11].

Preclinical evidence concerning JIB-04 in HNSCC has expanded in recent years. Dorna and colleagues evaluated several KDM-directed compounds, including JIB-04, in FaDu and SCC-040 cells treated with cisplatin and/or the PARP inhibitor olaparib. JIB-04 showed substantial single-agent activity, while the responses to combination treatment differed between the two cell lines. The study also reported pronounced effects of regimens incorporating all three agents [12]. In a separate study, JIB-04 combined with the histone deacetylase inhibitor panobinostat reduced viability in FaDu and SCC-152 cells and was associated with changes in apoptosis- and DNA damage-related readouts. More recently, Dorna and colleagues examined the effects of KDM-directed compounds, including JIB-04, on HNSCC cell growth and cisplatin response in two- and three-dimensional cultures [13]. Together, these studies establish a rationale for investigating JIB-04-containing combinations in HNSCC, while showing that treatment responses cannot be assumed to be uniform across cell models.

These previous studies did not examine the matched UT-SCC-24A/24B and UT-SCC-42A/42B cell-line pairs used here. Moreover, although functional outcomes and selected molecular markers have been investigated, a broader characterization of the early transcriptional response could help identify stress- and cell-cycle-related programs associated with JIB-04 treatment in the context of cisplatin exposure. We therefore compared the effects of JIB-04 and cisplatin across these established cell lines originating from primary tumors and matched lymph-node metastases. In UT-SCC-24A, selected for further study on the basis of a localized positive interaction in the Highest Single Agent analysis, we combined functional assays with RNA sequencing performed after 24 h of treatment. This approach allowed us to examine whether early changes in gene expression were consistent with the later effects on DNA synthesis, cell-cycle distribution, and apoptosis, and whether the combination elicited transcriptional effects beyond those associated with the individual treatments. The resulting associations were intended to generate mechanistic hypotheses rather than establish a causal role for any transcriptional pathway or demonstrate inhibition of a specific KDM.

## 2. Materials and Methods

### 2.1. Cell Lines and Cell Culture Conditions

The study included four established UT-SCC cell lines obtained from Turku University Central Hospital, Finland: UT-SCC-24A, established from a primary tongue squamous cell carcinoma; UT-SCC-24B, from a cervical lymph-node metastasis of the same patient; UT-SCC-42A, from a primary laryngeal squamous cell carcinoma; and UT-SCC-42B, from a cervical lymph-node metastasis of the patient from whom UT-SCC-42A was established (Supplementary Table S1). All four cell lines represent HPV-negative HNSCC models [14,15,16]. BJ normal human skin fibroblast cell line (CRL-2522) was obtained from the American Type Culture Collection (ATCC) (Rockville, MD, USA). All cell lines used in this study were tested for mycoplasma contamination and confirmed to be negative.

All cell lines were cultured in DMEM/F12 (Gibco, Grand Island, NY, USA) supplemented with 10% fetal bovine serum, 100 µg/mL streptomycin and 100 IU/mL penicillin (Gibco) at 37 °C in a humidified CO_2_ incubator (95% relative humidity). Cell density was determined prior to each experiment using an automated TC20™ cell counter (Bio-Rad, Hercules, CA, USA).

### 2.2. Drugs and Chemical Reagents

Cisplatin (Cat. HY-17394) and JIB-04 (Cat. HY-13953) were purchased from MedChemExpress (Monmouth Junction, NJ, USA). Cisplatin was dissolved in sterile 0.9% sodium chloride (NaCl) solution according to the manufacturer’s recommendations to prepare a stock solution; aliquots were stored at −20 °C protected from light and used within one month. JIB-04 stock solutions were prepared in DMSO according to the manufacturer’s recommendations and stored at −80 °C until use. To achieve the desired final concentrations, the stock solutions were further diluted in DMEM/F12 culture medium. The final concentration of DMSO in all treatment conditions did not exceed 0.1%, a level at which no cytotoxic effect on cell viability was observed.

### 2.3. Cell Viability Assessment

Cell viability was assessed using the MTT assay (Sigma-Aldrich, Saint Louis, MO, USA). Briefly, cells were seeded in 96-well plates at a density of 5 × 10^4^ cells/mL and allowed to attach and grow until they reached approximately 70–80% confluence (24 h). The next day, the culture medium was removed and the cells were treated with different concentrations of cisplatin and JIB-04 (individually or in combinations). After 72 h of incubation with the drugs, the medium was supplemented with MTT solution (3-(4,5-dimethylthiazol-2-yl)-2,5-diphenyltetrazolium bromide; 5 mg/mL in PBS) and the cells were incubated for 3h at 37 °C to allow formation of formazan crystals. Subsequently, the medium containing MTT was removed and the formazan crystals were dissolved in 200 µL of DMSO. Absorbance was measured at 570 nm using an Infinite M200 PRO microplate reader (Tecan, Männedorf, Switzerland) and cell viability was expressed as a percentage of the untreated control (mean ± SD). Each assay was conducted three times and was measured in triplicate. The IC_50_ values were determined using the AAT Bioquest IC_50_ calculator [AAT Bioquest, Inc. (6 June 2026). Quest Graph™ IC50 Calculator. AAT Bioquest. https://www.aatbio.com/tools/ic50-calculator accessed on 6 June 2026].

### 2.4. Evaluation of Cisplatin and JIB-04 Drug Combination

Drug interactions were evaluated in UT-SCC-24A, UT-SCC-24B, UT-SCC-42A, and UT-SCC-42B cells using dose–response matrices comprising JIB-04 and cisplatin administered individually and in combination. Following 72 h of treatment, cell viability was measured by the MTT assay, as described in Section 2.3, and expressed relative to the corresponding untreated control. The resulting dose–response data were analyzed using SynergyFinder 3.0 and the Highest Single Agent (HSA) reference model [17].

Under the HSA model, the observed effect of each drug combination is compared with the effect of the more active single agent at the corresponding concentrations. HSA interaction scores were calculated across each dose matrix and visualized as heat maps and three-dimensional interaction landscapes. For descriptive interpretation of HSA scores, values > 10 were classified as synergistic, values from 0 to 10 as additive, and values < 0 as antagonistic. Thus, positive scores indicate that the observed effect of the combination exceeded the HSA reference expectation, whereas negative scores indicate an effect lower than expected under the HSA model. Because interaction scores varied across the dose matrices, both overall HSA scores and the distribution of local scores were considered.

For subsequent functional and molecular analyses, UT-SCC-24A cells were treated with 0.075 µM JIB-04 and 6.935 µM cisplatin. This concentration pair was located within a narrow region of positive local HSA scores, with local scores above 10. The lower cisplatin concentration from this region was selected to avoid a pronounced effect of cisplatin alone and to retain a measurable response range for assessing the additional effects of co-treatment. BrdU incorporation, LDH release, cell-cycle distribution, apoptosis and gene-expression analyses performed at this concentration pair were not used to calculate drug-interaction scores.

### 2.5. Cell Proliferation Assay (ELISA BrdU)

The proliferation activity of UT-SCC-24A cell line after drug treatment was determined using the Cell Proliferation ELISA, 5-bromo-2-deoxyuridine (BrdU) Kit (Cat. 11647229001; Roche Diagnostics, Mannheim, Germany). First, the UT-SCC-24A cells were seeded at a density of  5 × 10^4^ cells/mL on the 96-well plate (200 µL per well) and treated for 72h with cisplatin and JIB-04, either alone or combined with the indicated concentrations of the tested compounds. After drug incubation, BrdU labeling solution was added to the cell culture medium to a final concentration of 10 μM BrdU and then incubated for 2 h to allow it to incorporate into newly synthesized DNA. The labeling medium was then removed and the cells were dried at 60 °C for 1 h. Afterwards, the cells were fixed for 30 min using the FixDenat solution provided with the kit. FixDenat was then removed, Anti-BrdU-POD working solution coupled with horseradish peroxidase was added and incubated for 90 min at RT, followed by the addition of tetramethylbenzidine substrate (TMB) (100 μL/well). The color development was stopped by adding 1 M sulphuric acid (H_2_SO_4_) and absorbance was measured at 450 nm using an Infinite M200 PRO microplate reader (Tecan, Männedorf, Switzerland).

### 2.6. LDH Cytotoxicity Assay

Cytotoxicity was assessed by measuring lactate dehydrogenase (LDH) release using the CyQUANT™ LDH Cytotoxicity Assay Kit (Thermo Fisher Scientific, Waltham, MA, USA). Cells were plated in 96-well plates at a density of 5 × 10^4^ cells/mL in 200 μL of complete culture medium and allowed to attach for 24 h. The cells were then treated with the indicated concentrations of the tested compounds in culture medium supplemented with 2% FBS. After 72 h of treatment, cell-culture supernatants were collected and analyzed according to the manufacturer’s protocol. Absorbance was measured at 490 nm and 680 nm using an Infinite M200 PRO microplate reader (Tecan). For each well, the background-corrected LDH signal was determined. Cytotoxicity was expressed as the percentage of LDH release relative to the maximum LDH release from lysed untreated control cells, after correction for spontaneous LDH release and cell-free medium background.

### 2.7. The Cell Cycle Assessment

The cell cycle phases at the single cell level were measured through the image analysis system NC-3000, using NC-3000 Two-step Cell Cycle Analysis Protocol (Chemometec, Allerød, Denmark), as described previously [18]. The UT-SCC-24A cells were seeded at a density of  5 × 10^4^ cells/mL into 6-well plates. The next day, cells were treated with JIB-04, cisplatin, DMSO as vehicle or a combination of both compounds—JIB-04 and cisplatin. After 72 h of treatment, the culture supernatant containing detached cells was collected. The remaining adherent cells were detached using trypsin–EDTA and neutralized with the previously collected culture supernatant. The combined cell suspension was centrifuged, and the resulting cell pellet was washed twice with phosphate-buffered saline. The pellet was then resuspended in 250 µL of lysis buffer (Solution 10) enriched with 10 µg/mL DAPI and incubated for 5 min at 37 °C. After adding 100 µL of Solution 11, a 10 µL aliquot was loaded onto an NC-Slide and analyzed using the NucleoCounter NC-3000.

### 2.8. Detection of Apoptosis

The apoptosis detection was performed using Annexin V/propidium iodide (PI) staining and the NucleoCounter NC-3000 system (Denmark, Chemometec). The UT-SCC-24A cells were seeded at a density of  5 × 10^4^ cells/mL into 6-well plates. The next day, cells were treated with JIB-04, cisplatin, DMSO as vehicle or a combination of both compounds—JIB-04 and cisplatin. After 72 h of treatment, the culture supernatant containing detached cells was collected. The remaining adherent cells were detached using trypsin–EDTA and neutralized with the previously collected culture supernatant. The combined cell suspension was centrifuged, and the resulting cell pellet was washed twice with phosphate-buffered saline. Cells were resuspended in 100 µL of Annexin V binding buffer and gently mixed with 2 µL of Annexin V-CF488A conjugate and 4 µL of Hoechst 33342 (10 µg/mL). Following incubation at 37 °C for 15 min, samples were centrifuged at 400× *g* for 5 min. The pellets were resuspended in 100 µL of binding buffer containing PI (10 µg/mL), prepared by adding 2 µL of Solution 16 to 100 µL of binding buffer. A 30 µL aliquot was loaded onto an NC-Slide for analysis using the NucleoCounter NC-3000.

### 2.9. Quantitative PCR Analysis

The expression levels of mRNA were measured using qPCR. The UT-SCC-24A cells were seeded at a density of  5 × 10^4^ cells/mL in 6-well plates. The next day, cells were treated with JIB-04, cisplatin, DMSO as vehicle or a combination of both compounds—JIB-04 and cisplatin. After 72 h of incubation with tested compounds, total RNA was extracted using RNeasy Mini Kit (Qiagen, Hilden, Germany) following the manufacturer’s instructions. The quality of total RNA isolated was assessed by agarose gel electrophoresis and the quantity of RNA was measured using an Infinite M200 PRO microplate reader (Tecan). Complementary DNA (cDNA) was obtained from an RNA template using High-Capacity cDNA Reverse Transcription Kit (Applied Biosystems, Foster City, CA, USA). The qPCR was performed in 96-well plate format with the LightCycler^®^ 480 II instrument (Roche, Basel, Switzerland) using PowerUp SYBR Green Master Mix (Applied Biosystems) protocol. Relative mRNA expression was calculated using the subtraction method (relative quantification, RQ = 2^−∆∆Ct^) and normalized to the expression of the *GAPDH/ACTB* housekeeping genes. The following primer sequences were used and are presented in Table 1.

### 2.10. RNA Sequencing and Bioinformatic Analysis

An earlier RNA-seq dataset generated from untreated UT-SCC-24A, UT-SCC-24B, UT-SCC-42A, and UT-SCC-42B cells was used for descriptive assessment of basal lysine demethylase gene expression across the cell-line panel. This analysis was independent of the subsequent RNA-seq experiment performed in UT-SCC-24A cells after treatment with JIB-04, cisplatin, or their combination. Expression values for selected KDM genes were visualized using regularized-logarithm (rlog)-transformed data (Supplementary Figure S1).

For the treatment-response RNA-seq experiment, UT-SCC-24A cells were seeded in 6-well plates and allowed to attach for 24 h. Cells were subsequently treated for 24 h with vehicle control, 0.075 µM JIB-04, 6.935 µM cisplatin, or their combination, using the same drug concentrations as in the functional assays. Total RNA was isolated using the RNeasy Mini Kit (Qiagen, Hilden, Germany) according to the manufacturer’s instructions. Three independent biological replicates were analyzed for each treatment condition. Paired-end sequencing data generated across two NovaSeq X flow cells were processed as described in the Supplementary Methods.

Differential expression was analyzed with DESeq2 (version 1.46.0) using a negative binomial generalized linear model with one four-level factor (design~treatment; *n* = 3 biological replicates per group) and Wald tests. In addition to each treatment versus control and the combination versus each single drug, the drug interaction was tested as the contrast log2FC(JIB-04 + cisplatin vs. control) − log2FC(JIB-04 vs. control) − log2FC(cisplatin vs. control), which equals zero when the effects of the two drugs are additive on the log2 expression scale (i.e., multiplicative, independent effects on the linear scale). *p*-values were adjusted for multiple testing with the Benjamini–Hochberg method, with DESeq2 independent filtering (alpha = 0.05); genes with *p*_adj_ < 0.05 were considered significant and, where indicated, an additional threshold of |log2FC| > 1 (unshrunken estimates) was applied. Genes with a significant interaction (*p*_adj_ < 0.05) were classified, using unshrunken log2FC, as greater-than-additive (|observed| > |expected| with the same sign), less-than-additive (|observed| < |expected|, same sign) or opposite to the additive expectation (different sign), where observed is the log2FC of the combination and expected = log2FC(JIB-04) + log2FC(cisplatin). These terms describe gene expression on the log2 scale only and are distinct from pharmacological synergy, which was assessed separately from cell viability data using the HSA model. Gene set enrichment analysis (clusterProfiler/fgsea) was performed on all genes ranked by the DESeq2 Wald statistic of each contrast, including the interaction contrast, with Benjamini–Hochberg adjustment. For the principal component analysis (variance-stabilized data, 500 most variable genes), the position expected for the combination under additivity was calculated as centroid(JIB-04) + centroid(cisplatin) − centroid(control), and the deviation in the observed combination centroid from this position was expressed as a percentage of the length of the control-to-combination vector; this descriptive measure was not used for statistical inference.

### 2.11. Statistical Analysis and Preparation of Graphs

Statistical analyses of the functional and qPCR experiments were performed using GraphPad Prism 10.2.2 for Windows (GraphPad Software, Boston, MA, USA). RNA-seq analyses were performed in R (version 4.4.1), as described in Section 2.10 and Supplementary Methods S1. Exploratory Western blot densitometry was analyzed in Python using SciPy (SciPy, version 1.18.1), as described in Supplementary Methods S2. For concentration–response experiments, multiple treatment concentrations were compared with the vehicle control using one-way ANOVA followed by Dunnett’s multiple-comparisons test. For experiments involving pairwise comparisons among all treatment groups, one-way ANOVA followed by Tukey’s multiple-comparisons test was used (* *p* ≤ 0.05; ** *p* ≤ 0.01; *** *p* ≤ 0.001 **** *p* ≤ 0.0001 was considered statistically significant).

Each experiment was performed independently three times using independently prepared cell cultures. Within each independent experiment, each treatment condition in the functional and qPCR assays was measured in three technical replicates. Technical replicates were averaged before statistical analysis; thus, the experimental unit was the independent biological experiment (*n* = 3, unless otherwise specified; the numbers of valid biological replicates for Western blot densitometry are given in Supplementary Figure S4 and Supplementary Methods S2).

## 3. Results

### 3.1. JIB-04 and Cisplatin Reduce Cell Viability in HNSCC Cells

**Figure 1:**
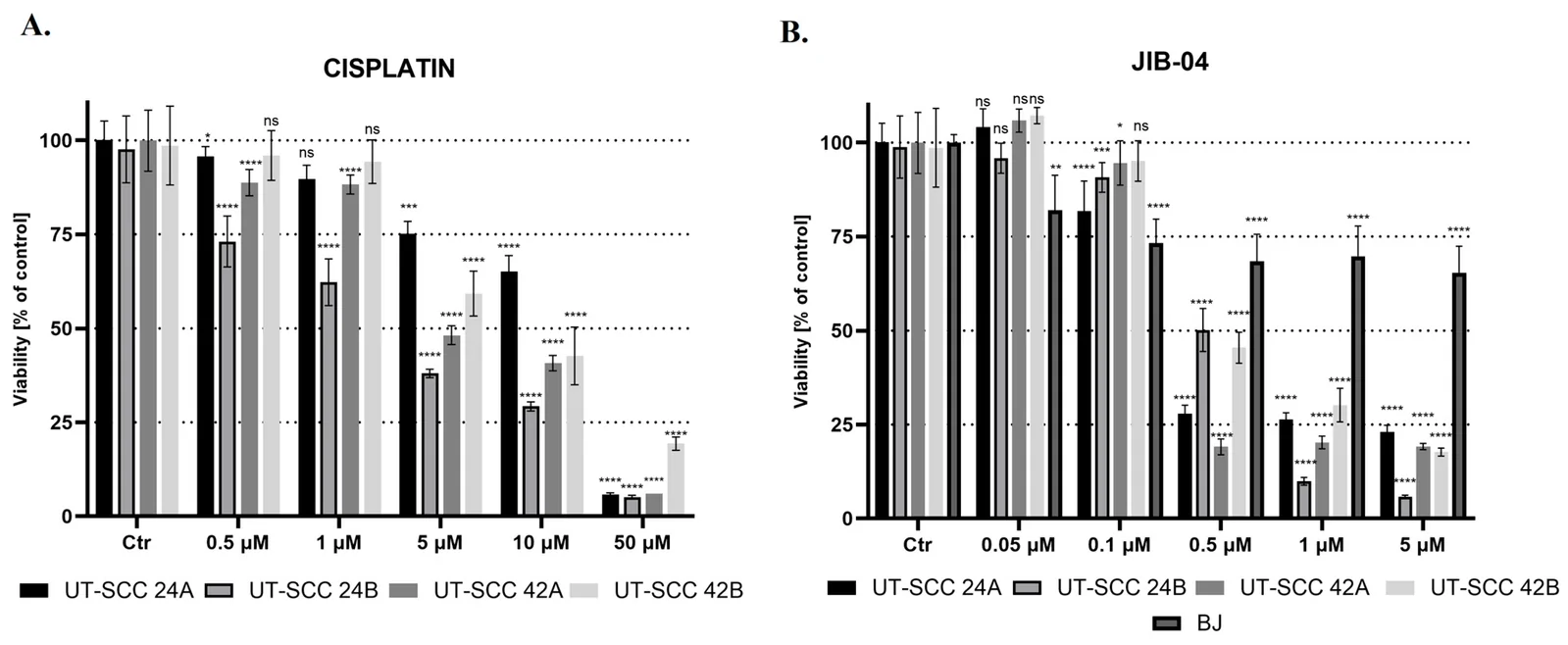
Effects of cisplatin and JIB-04 on the MTT response of established HNSCC cell lines. UT-SCC-24A, UT-SCC-24B, UT-SCC-42A, and UT-SCC-42B cells were treated for 72 h with increasing concentrations of (**A**) cisplatin (0.5–50 µM) or (**B**) JIB-04 (0.05–5 µM). BJ human skin fibroblasts were included in the JIB-04 dose–response analysis. The MTT signal was normalized to the control. Both compounds produced concentration-dependent reductions, with estimated IC_50_ values of 13.87 µM for cisplatin and 0.30 µM for JIB-04 in UT-SCC-24A cells. The BJ fibroblast data are presented as an exploratory comparison and do not establish tumor-cell selectivity. Data are presented as mean ± SD from three independent experiments. Comparisons with the control were performed using one-way ANOVA followed by Dunnett’s test: ns, *p* ≥ 0.05; *, *p* < 0.05; **, *p* < 0.01; ***, *p* < 0.001; ****, *p* < 0.0001.

The initial screening studies were performed in four HNSCC cell lines (UT-SCC-24A, UT-SCC-24B, UT-SCC-42A, UT-SCC-42B) to determine their sensitivity to cisplatin and the histone demethylase inhibitor JIB-04, as well as to select appropriate concentrations for subsequent combination experiments. Furthermore, JIB-04 was also tested on normal BJ fibroblasts. Both agents were evaluated across a wide range of doses using MTT viability assays after 72 h of treatment (Figure 1).

Dose–response curves for cisplatin showed a concentration-dependent decrease in viability in all cell lines, with UT-SCC-24A being the most resistant (IC_50_ = 13.87 µM), while the most pronounced decrease in viability at lower concentrations was observed in the UT-SCC-24B cell line. In contrast, JIB-04 demonstrated a dose-dependent reduction in cell survival across all tumor cell lines, even at submicromolar concentrations, with UT-SCC-24B being the most sensitive.

For UT-SCC-24A cells, the IC_50_ for JIB-04 was estimated at 0.3 µM. JIB-04 was also tested in BJ skin fibroblasts, in which the observed response was less pronounced under the conditions examined (Figure 1B). Because BJ fibroblasts are not tissue-matched epithelial controls, these data were not used to infer tumor selectivity.

### 3.2. Combination Analysis Identifies a Localized Positive HSA Interaction

**Figure 2:**
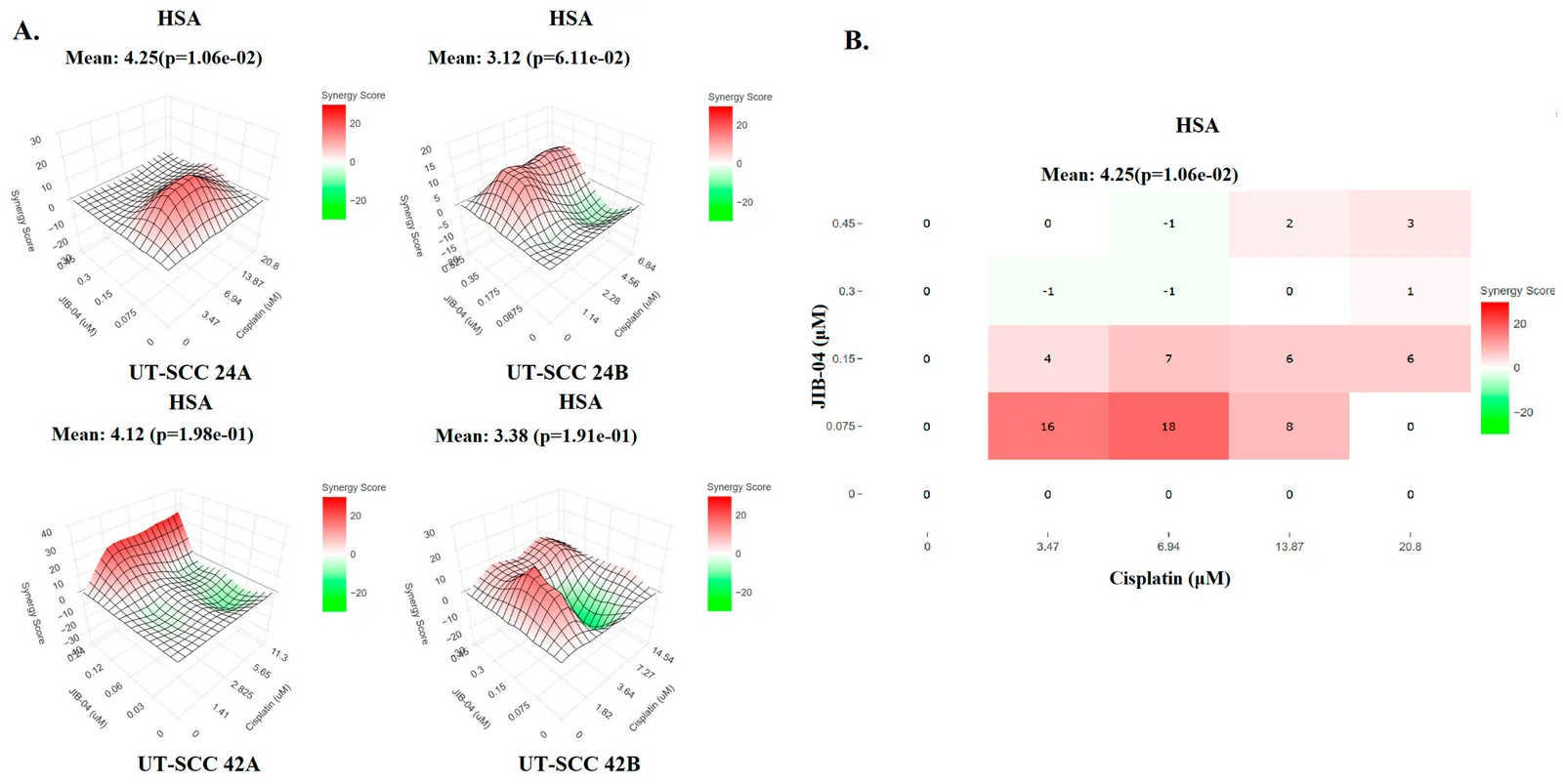
Dose-dependent interaction between JIB-04 and cisplatin in HNSCC cell lines. (**A**) UT-SCC-24A, UT-SCC-24B, UT-SCC-42A, and UT-SCC-42B cells were treated for 72 h with increasing concentrations of cisplatin and JIB-04, alone or in combination. Cell viability was assessed by MTT assay, and the dose–response data were analyzed using SynergyFinder 3.0 with the Highest Single Agent (HSA) reference model. Three-dimensional plots show the distribution of HSA interaction scores across the tested concentrations. Although the mean HSA scores were positive in all four cell lines, the interaction was most pronounced in UT-SCC-24A. (**B**) The HSA interaction heat map for UT-SCC-24A shows a localized region of higher positive scores (approximately 16–18) at 0.075 µM JIB-04 combined with 6.935-13.87 µM cisplatin.

We assessed the combined effects of JIB-04 and cisplatin across a concentration matrix in each UT-SCC cell line (Figure 2). MTT responses were analyzed using SynergyFinder 3.0 and the Highest Single Agent (HSA) reference model, which compares the effect of each combination with that of the more active single agent at the corresponding concentrations. Heat maps and three-dimensional interaction landscapes were used to visualize HSA scores across the matrices. In UT-SCC-24A, the overall HSA score was 4.25, reflecting a modest positive interaction averaged across the tested concentrations. The effect was concentrated within a narrow region, where local HSA scores reached approximately 16–18 at 0.075 µM JIB-04 combined with 6.935–13.87 µM cisplatin. We therefore describe the response as a localized positive interaction rather than a broadly enhanced effect across the matrix. For subsequent analyses, we selected 0.075 µM JIB-04 with 6.935 µM cisplatin, the lower cisplatin concentration within this region, to retain a measurable range for assessing the effects of co-treatment.

### 3.3. Co-Treatment Further Reduces DNA Synthesis

To assess the impact of JIB-04 and cisplatin on proliferative capacity, DNA synthesis was quantified in UT-SCC-24A cells using a BrdU ELISA assay, which measures incorporation of the thymidine analog BrdU into newly synthesized DNA in actively proliferating cells. In line with the interaction analysis, treatment with either 0.075 µM JIB-04 or 6.935 µM cisplatin alone significantly reduced BrdU incorporation.

**Figure 3:**
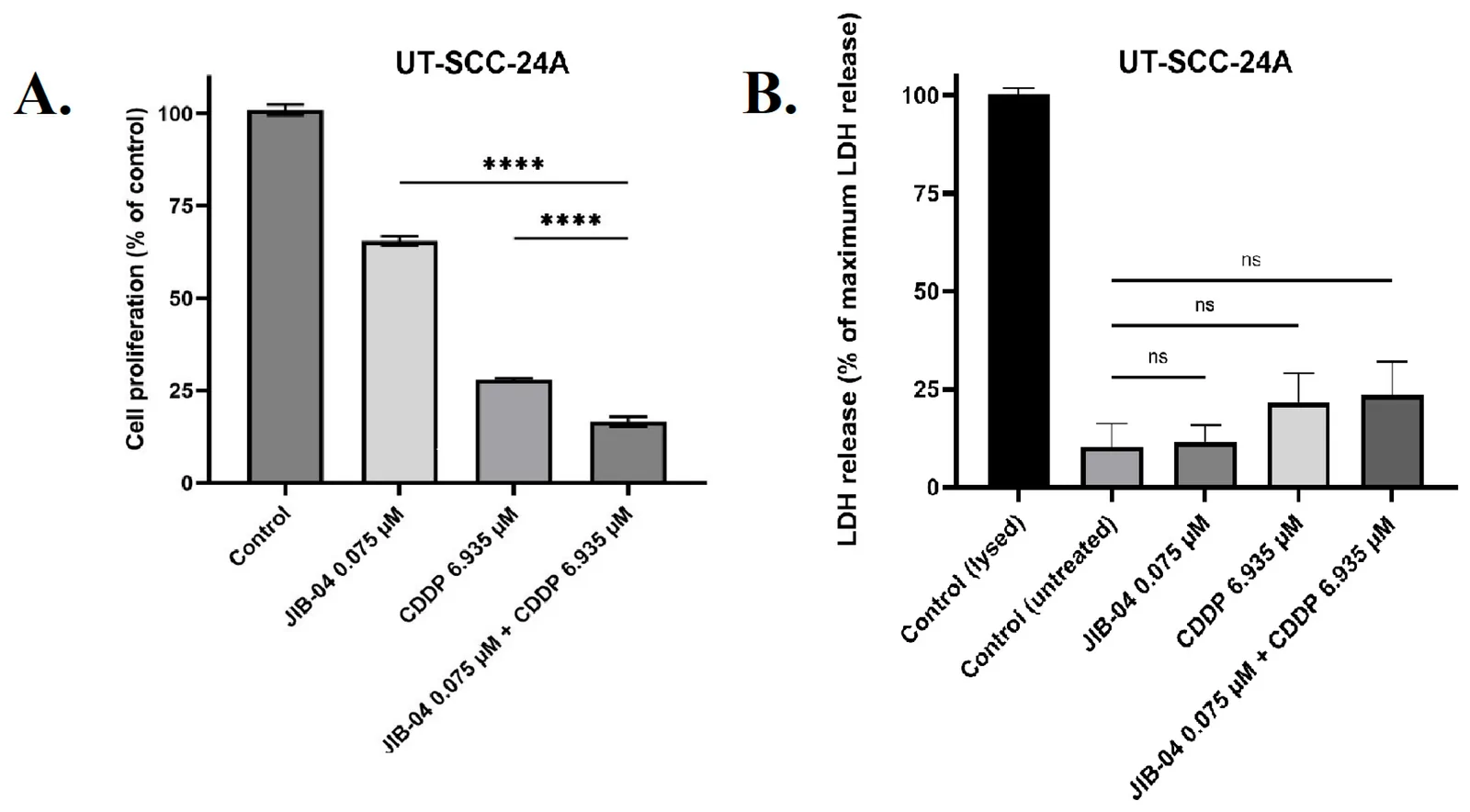
Effects of JIB-04 and cisplatin co-treatment on DNA synthesis and LDH release in UT-SCC-24A cells. (**A**) BrdU incorporation was measured after 72 h of treatment with 0.075 µM JIB-04, 6.935 µM cisplatin, or their combination. Co-treatment reduced BrdU incorporation more than either single agent. (**B**) LDH release was measured after 72 h under the same treatment conditions and expressed as a percentage of the maximum release from lysed control cells. Data are presented as mean ± SD from three independent experiments. Statistical analysis was performed using one-way ANOVA followed by Tukey’s post hoc test; ns, not significant (*p* ≥ 0.05; ****, *p* < 0.0001.).

In UT-SCC-24A cells, treatment with either 0.075 µM JIB-04 or 6.935 µM cisplatin for 72 h reduced BrdU incorporation compared with the control (Figure 3A). Co-treatment further decreased BrdU incorporation relative to both single-agent treatments, indicating greater suppression of DNA synthesis at the selected concentrations.

### 3.4. JIB-04 and Cisplatin Co-Treatment Does Not Significantly Increase LDH Release

LDH release was assessed in UT-SCC-24A cells after 72 h of treatment (Figure 3B). No statistically significant difference was detected between the control and the JIB-04, cisplatin, or combination groups. Although the measured LDH release was numerically higher following cisplatin and combination treatment than in the control group, these differences did not reach statistical significance. Thus, the assay did not provide evidence of increased LDH release under the tested conditions; this result should not be interpreted as proof of an absence of membrane damage.

### 3.5. JIB-04 and Cisplatin Co-Treatment Alters Cell Cycle Distribution in UT-SCC-24A Cells

**Figure 4:**
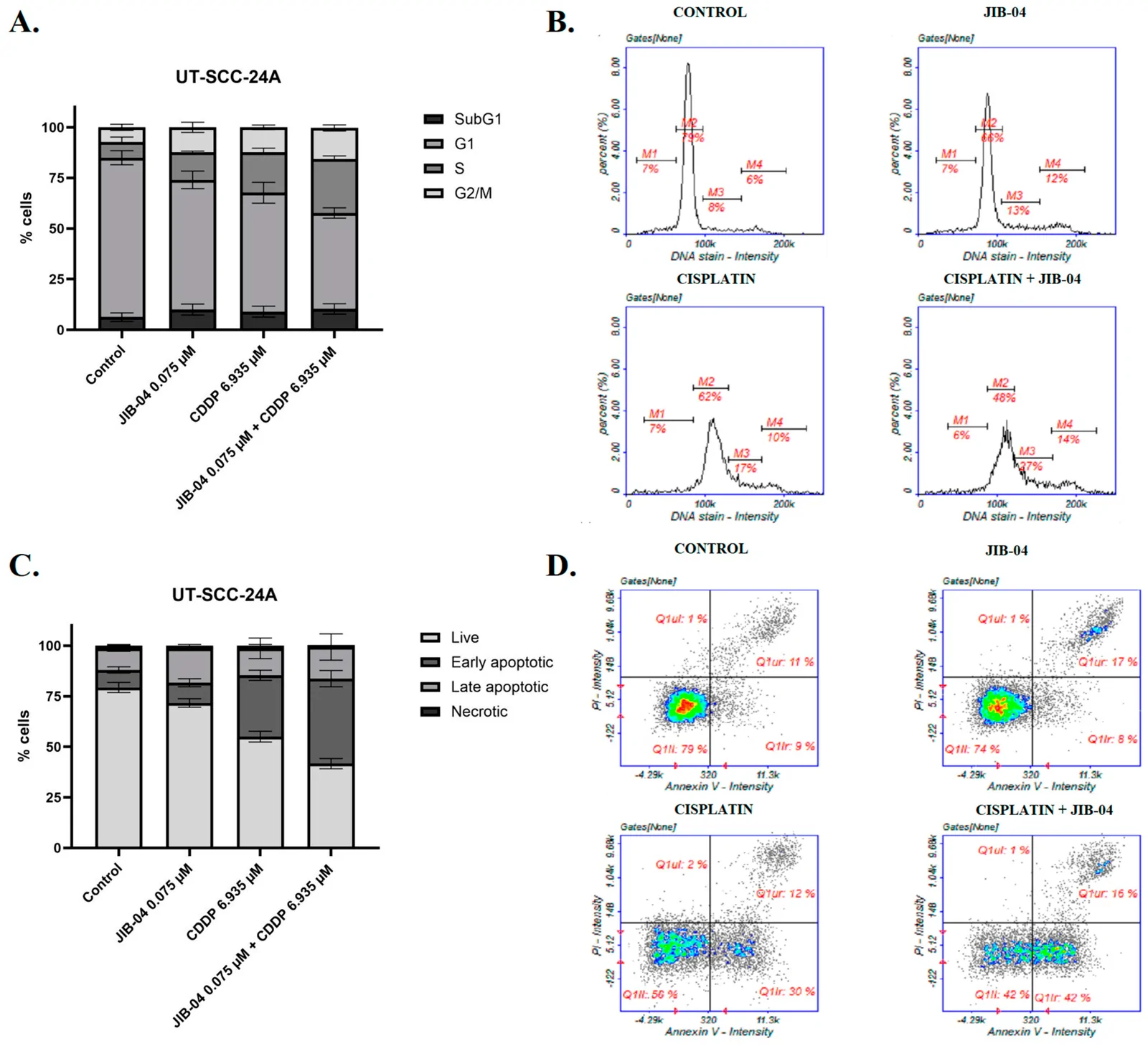
Effects of JIB-04 and cisplatin co-treatment on cell cycle distribution and apoptosis in UT-SCC-24A cells. (**A**) Quantification of cells in each cell cycle phase and (**B**) representative cell cycle histograms after 72 h of treatment. JIB-04 alone increased the proportion of cells in the G2/M phase, whereas cisplatin increased the proportions in the S and G2/M phases. Co-treatment was associated with higher S- and G2/M-phase proportions and a lower G1-phase proportion than cisplatin alone. (**C**) Quantification of viable, early apoptotic, and late apoptotic cells and (**D**) representative Annexin V/PI dot plots. Both cisplatin and JIB-04 alone increased the proportions of apoptotic cells compared with the untreated control; notably, JIB-04 alone increased the proportion of late apoptotic cells. Co-treatment was associated with a lower proportion of viable cells and a higher overall apoptotic fraction than either single-agent treatment. Data are presented as mean ± SD from three independent experiments, each performed in triplicate.

To gain insight into the mechanism of action of cisplatin and JIB-04, as well as to assess their potential synergistic interaction, we next analyzed cell cycle distribution in UT-SCC-24A cells. Cell cycle analysis showed that JIB-04 alone caused an approximately two-fold increase in the fraction of cells in the G2/M phase compared with the untreated control, indicating an inhibition of cell cycle progression at this checkpoint. Cisplatin monotherapy induced a characteristic redistribution of cells with a pronounced accumulation in the S and G2/M phases, which is consistent with the induction of DNA damage and activation of S/G2-phase checkpoints. Importantly, the combined treatment with cisplatin and JIB-04 further intensified the cisplatin-induced changes, leading to a more pronounced increase in the S and G2/M populations and a concomitant reduction in cells in the G1 phase (Figure 4A,B). These findings show that, at the selected concentrations, co-treatment was associated with a more pronounced alteration in cell-cycle distribution than either single-agent treatment in UT-SCC-24A cells.

### 3.6. Combined JIB-04 and Cisplatin Treatment Increases Apoptosis in UT-SCC-24A Cells

In UT-SCC-24A cells, Annexin V/PI staining showed that treatment with either cisplatin or JIB-04 alone increased the proportions of early and late apoptotic cells compared with the untreated control. In particular, JIB-04 alone was associated with an increased late apoptotic fraction. Co-treatment was associated with a lower proportion of viable cells and a higher proportion of Annexin V-positive cells than either single-agent treatment (Figure 4C,D). These findings indicate a greater apoptotic response under the combination treatment at the concentrations tested.

### 3.7. JIB-04 and Cisplatin Co-Treatment Alters Apoptosis- and EMT-Related Gene Expression in UT-SCC-24A Cells

**Figure 5:**
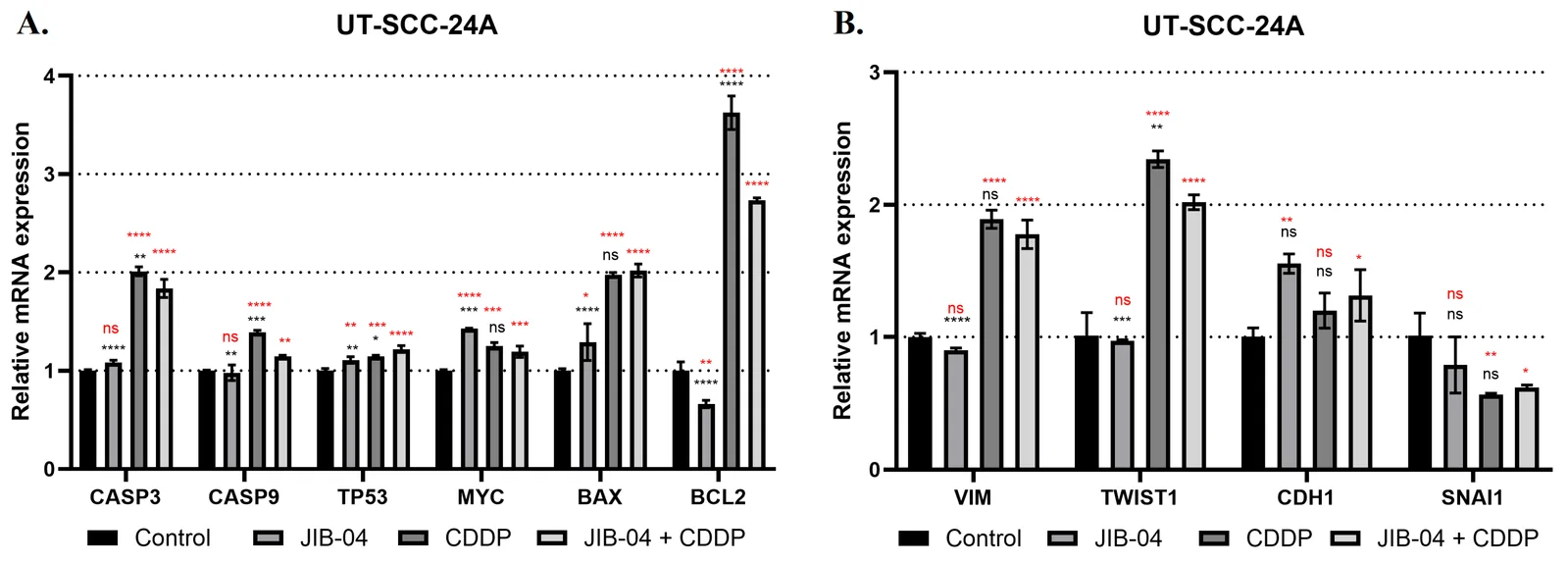
Expression of apoptosis- and EMT-related genes in UT-SCC-24A cells after treatment with JIB-04 and cisplatin. (**A**) qPCR analysis of *CASP3*, *CASP9*, *BAX*, and *BCL2* expression. Cisplatin increased the mRNA levels of all four genes relative to the untreated control, including an approximately two-fold increase in *BAX* and a 3.5–4-fold increase in *BCL2*. Under co-treatment, *BAX* expression was similar to that observed with cisplatin alone, whereas *BCL2* expression was lower. JIB-04 alone produced smaller changes in the expression of the genes analyzed. (**B**) qPCR analysis of the EMT-related genes *CDH1*, *VIM*, *TWIST1*, and *SNAI1*. Cisplatin increased *VIM* and *TWIST1* expression, while JIB-04 alone slightly increased *CDH1* expression and was associated with lower VIM and *SNAI1* expression. *VIM* and *TWIST1* expression remained elevated relative to the untreated control under co-treatment. Data are presented as mean ± SD from three independent experiments. Statistical significance was assessed by one-way ANOVA followed by Tukey’s post hoc test. Red asterisks indicate comparisons with the untreated control; black asterisks indicate comparisons of the combination with the indicated single-agent treatment (cisplatin or JIB-04). ns, *p* ≥ 0.05; *, *p* < 0.05; **, *p* < 0.01; ***, *p* < 0.001; ****, *p* < 0.0001.

To further characterize transcriptional changes associated with treatment, we assessed the expression of selected EMT- and apoptosis-related genes in UT-SCC-24A cells treated with JIB-04, cisplatin, or their combination (Figure 5). Cisplatin treatment was associated with increased *VIM* and *TWIST1* mRNA expression, modest changes in *CDH1* expression, and reduced *SNAI1* expression compared with the untreated control. JIB-04 alone was associated with a slight increase in *CDH1* expression and lower *VIM* and *SNAI1* mRNA levels. Under co-treatment, *VIM* and *TWIST1* expression remained elevated relative to the untreated control. These changes indicate treatment-associated differences in the expression of selected EMT-related transcripts but do not establish EMT induction or reversal at the phenotypic level.

Cisplatin increased *CASP3*, *CASP9*, *BAX*, and *BCL2* mRNA expression. *BAX* expression was approximately two-fold higher, whereas *BCL2* expression was approximately 3.5–4-fold higher than in untreated control cells. In the co-treatment group, *BAX* mRNA levels remained similar to those observed after cisplatin treatment alone, while *BCL2* mRNA expression was lower than after cisplatin treatment. JIB-04 alone had little effect on *CASP3* and *CASP9* transcript levels and was associated with a slight increase in *BAX* and a decrease in *BCL2* expression.

Overall, the lower *BCL2* expression observed in co-treated cells, together with sustained *BAX* expression, was associated with a more proapoptotic *BAX/BCL2* transcript ratio than that observed following cisplatin treatment alone. This expression pattern is consistent with the increased proportion of apoptotic cells detected by Annexin V/PI staining (Figure 4C,D). However, these qPCR findings do not demonstrate corresponding changes in protein abundance, establish activation of the intrinsic apoptotic pathway, or identify the molecular basis of the enhanced apoptotic response.

### 3.8. Early Transcriptional Responses to JIB-04, Cisplatin, and Their Combination in UT-SCC-24A Cells

To characterize early transcriptional responses before the 72 h functional end points, RNA-seq was performed in UT-SCC-24A cells after 24 h of treatment with JIB-04, cisplatin, or their combination.

The combination differed significantly from both JIB-04 and cisplatin alone (6358 and 5098 genes at *p*_adj_ < 0.05, and 1041 and 434 genes with |log2FC| > 1, respectively), with a profile dominated by the cisplatin response. In the PCA (Supplementary Figure S2), the combination was located relatively close to the position expected for additive effects of the two drugs, with a deviation corresponding to 18% of the length of the combination vector (a descriptive measure, unrelated to the variance explained by PC2), and the combination being displaced further along the cisplatin axis (PC1) than expected. At the gene level, a statistically significant drug interaction (*p* < 0.05) was detected for 894 of the 11,463 genes retained after independent filtering, 32 of which also showed an absolute interaction log2FC > 1; of the 766 differentially expressed genes for which the interaction could be evaluated, 600 (78.3%) had no statistically significant interaction and 166 (21.7%) showed a statistically significant interaction. A non-significant interaction test does not establish additivity; significant departures from the additive expectation were detected for a subset of genes and pathways (23 of 50 Hallmark gene sets). This transcriptional interaction analysis does not measure pharmacological synergy, which was evaluated separately from cell viability (HSA model).

**Figure 6:**
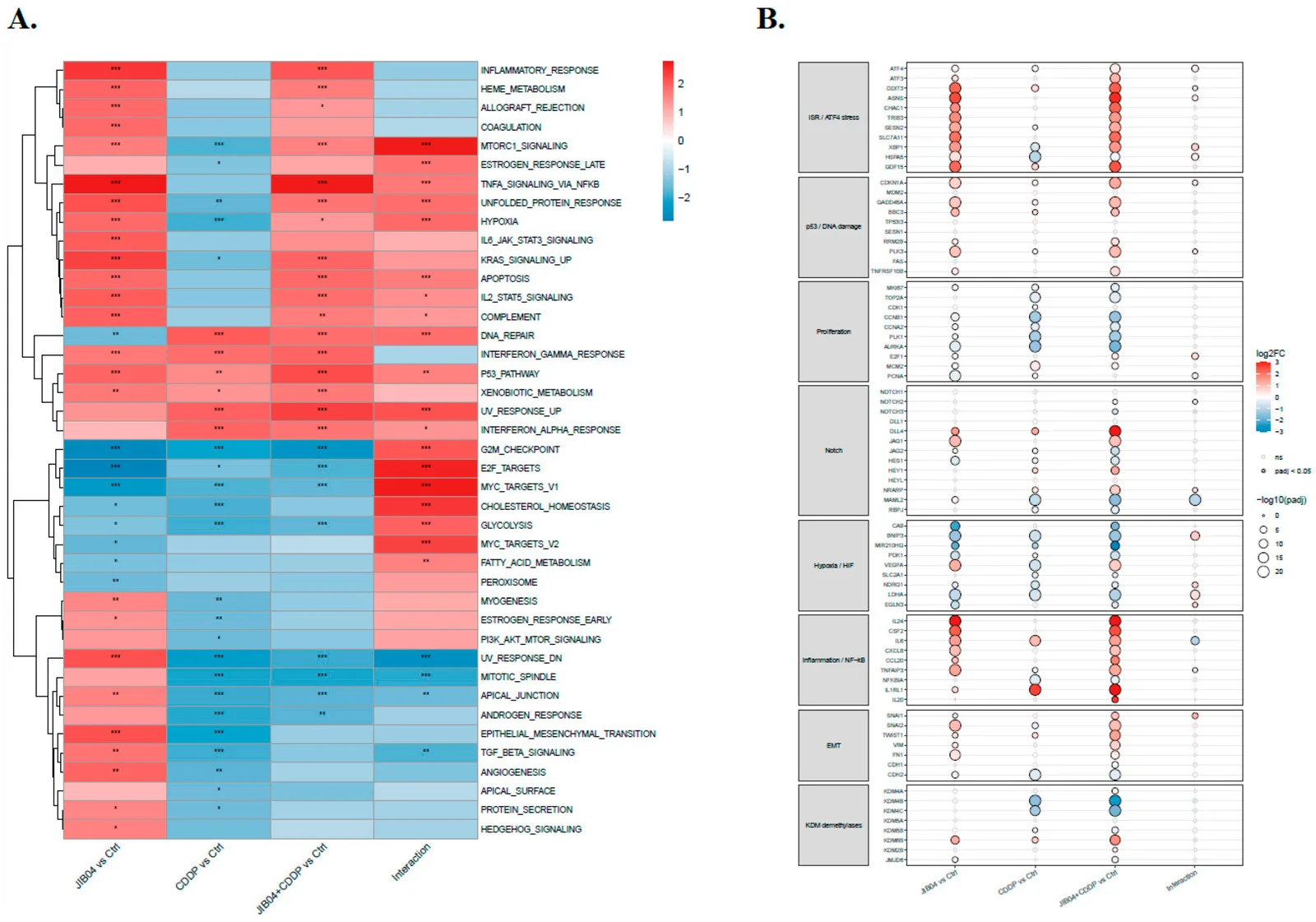
Transcriptomic responses to JIB-04, cisplatin, and their combination in UT-SCC-24A cells after 24 h of treatment. (**A**) Gene set enrichment analysis of MSigDB Hallmark pathways for JIB-04 versus control, cisplatin versus control, JIB-04 plus cisplatin versus control, and the transcriptomic interaction contrast. Normalized enrichment scores (NES) are shown. Positive and negative NES values indicate enrichment among genes upregulated and downregulated, respectively, in the indicated comparison. Asterisks indicate Benjamini–Hochberg adjusted *p*-values: *, *p*_adj_ < 0.05; **, *p*_adj_ < 0.01; ***, *p*_adj_ < 0.001. The interaction contrast represents the deviation in the observed combination response from the sum of the single-treatment effects on the log2 expression scale and was not used to calculate pharmacological drug-synergy scores. (**B**) Expression changes in selected genes representing integrated stress response/ATF4 signaling, p53/DNA damage response, proliferation, Notch signaling, hypoxia/HIF signaling, inflammation/NF-κB signaling, epithelial–mesenchymal transition, and lysine demethylases. Shrunken log2 fold changes are shown for treatment-versus-control comparisons; unshrunken log2 fold changes are shown for the interaction contrast. Black outlines indicate genes with *p*_adj_ < 0.05. RNA-seq was performed using three independent biological replicates per treatment group.

Gene set enrichment analysis showed that JIB-04 treatment was associated with activation of stress- and inflammatory-response programs, including TNFα signaling via NF-κB, inflammatory response, unfolded protein response, and hypoxia-related gene sets, together with reduced enrichment of proliferation-associated gene sets, including E2F targets, G2/M checkpoint, and MYC targets (Figure 6A). At the gene level, JIB-04 increased expression of integrated stress response/ATF4-associated genes, including *ASNS*, *DDIT3*, *GDF15*, *SLC7A11*, *TRIB3*, *CHAC1*, *XBP1*, and *SESN2* (Figure 6B).

Cisplatin treatment was associated with enrichment of DNA damage- and stress-related programs, including DNA repair, UV response, interferon responses, and the p53 pathway. In parallel, cisplatin reduced enrichment of G2/M checkpoint, mitotic spindle, and epithelial–mesenchymal transition gene sets (Figure 6A). Cisplatin also reduced the expression of several mitotic and proliferation-related genes, including *CCNB1*, *AURKA*, and *PLK1* (Figure 6B).

The combination treatment retained the JIB-04-associated integrated stress response and the cisplatin-associated DNA damage response. It was associated with activation of TNFα signaling via NF-κB, UV response, p53 pathway, inflammatory response, and interferon-γ response, together with reduced enrichment of G2/M checkpoint, mitotic spindle, UV response DN, and E2F target gene sets (Figure 6A). The expression of *ASNS*, *DDIT3*, and *GDF15* remained elevated under the combination treatment, while *CDKN1A* expression was higher than after either single-agent treatment (Figure 6B).

Overall, the RNA-seq analysis indicates that JIB-04 and cisplatin elicited distinct early transcriptional responses in UT-SCC-24A cells. The combination retained major features of both single-agent responses, including JIB-04-associated stress-response transcription and cisplatin-associated DNA damage and cell-cycle-related changes.

## 4. Discussion

The present study examined the effects of JIB-04 and cisplatin in four established UT-SCC cell lines originating from primary HNSCC tumors and matched lymph-node metastases. Both compounds reduced the MTT signal as single agents, although the magnitude and concentration dependence of the response differed among cell lines. Analysis of the dose matrices using the Highest Single Agent (HSA) model identified a modest positive overall interaction in UT-SCC-24A, with local HSA scores above 10 confined to a narrow concentration region comprising 0.075 µM JIB-04 and 6.935–13.87 µM cisplatin. In accordance with the predefined interpretation criteria, these local scores were classified as synergistic, whereas the overall HSA score of 4.25 indicated an additive interaction when averaged across the tested matrix. Therefore, the interaction should be interpreted as a localized positive, or locally synergistic, interaction rather than as a broad synergistic effect across the full dose matrix or across all HNSCC cell lines.

UT-SCC-24A was selected for subsequent analyses because it contained the concentration region with the highest positive HSA scores. The mechanistic experiments were intended to characterize cellular responses to this selected combination, rather than to establish a general mechanism of cisplatin sensitization across HNSCC models. Testing additional synergy-negative models would be valuable for determining whether the observed responses are specific to the interaction-positive context; however, these experiments were beyond the scope of the present study. Accordingly, the functional, molecular, and transcriptomic findings are limited to UT-SCC-24A cells.

At the selected concentrations, co-treatment was associated with a greater reduction in MTT signal and BrdU incorporation than either single-agent treatment. The BrdU assay shows reduced DNA synthesis under co-treatment but was not used to calculate an interaction score and therefore does not independently establish pharmacological synergy. Cell-cycle analysis showed that JIB-04 alone increased the G2/M population, whereas cisplatin increased the proportions of cells in the S and G2/M phases. Co-treatment was associated with a further increase in the S- and G2/M-phase populations and a reduction in the G1 population compared with cisplatin alone. Annexin V/PI staining showed a lower viable-cell fraction and a higher apoptotic-cell fraction after co-treatment than after either single agent. By contrast, LDH release was not significantly increased after 72 h. This result should not be interpreted as evidence that membrane damage was absent, but rather that increased LDH release was not detected under the conditions tested.

RNA-seq analysis after 24 h provided an earlier view of transcriptional responses, before the later 72 h functional end points. Principal component analysis showed clear separation of the treatment groups, with cisplatin accounting for the largest component of transcriptomic variation and JIB-04 producing a distinct secondary shift. JIB-04 and cisplatin were associated with different transcriptional programs. JIB-04 treatment induced an integrated stress response/ATF4-related signature, including increased expression of *ASNS*, *DDIT3*, *GDF15*, *SLC7A11*, *TRIB3*, *CHAC1*, *XBP1*, and *SESN2*, and was associated with enrichment of unfolded-protein-response and inflammatory-response gene sets. It was also associated with reduced enrichment of E2F targets, G2/M checkpoint, and MYC target gene sets. Cisplatin was associated with enrichment of DNA-repair, UV-response, interferon-response, and p53- associated gene sets, together with reduced enrichment of G2/M checkpoint and mitotic-spindle programs. The combination retained major features of both individual treatments, including the JIB-04-associated stress-response signature and cisplatin-associated DNA-damage and cell-cycle-related changes. In addition, *CDKN1A* expression was higher under co-treatment than after either single agent. *CDKN1A*, which encodes the cyclin-dependent kinase inhibitor p21, is a canonical p53 target involved in cell-cycle arrest. However, *CDKN1A* can also be induced through p53-independent stress-response pathways. Because UT-SCC-24A has been reported to carry a structurally disruptive *TP53* alteration, the increased *CDKN1A* expression observed under co-treatment should not be interpreted as evidence of canonical wild-type p53 activation or of a p53-dependent mechanism [14,15,16].

The transcriptomic interaction analysis should be interpreted separately from the pharmacological HSA analysis. For most genes for which interaction testing was possible, no statistically significant departure from the additive expectation on the log2 expression scale was detected; this absence of statistical significance does not establish additivity. A subset of genes showed expression in the combination that differed from the sum of the single-treatment effects. Globally, the combination transcriptome was dominated by the cisplatin-associated response while retaining the JIB-04-associated stress-response program. Therefore, the RNA-seq analysis does not support broad transcriptional synergy. Instead, it identifies treatment-associated transcriptional changes and selected deviations from additivity that may guide future functional studies. In particular, the p53-pathway and UV-response signatures were more strongly enriched under co-treatment than under either single-agent treatment, whereas several proliferation- and biosynthetic programs showed less suppression than predicted by additivity. These transcriptional patterns require independent validation and should not be equated with pharmacological synergy.

Cisplatin treatment and co-treatment were also associated with preferential downregulation of long genes. Cisplatin-induced DNA lesions can interfere with RNA polymerase II progression, such that long transcripts may be particularly susceptible to incomplete transcription. In the present dataset, many of the apparent neuronal and synaptic Gene Ontology terms among cisplatin-repressed genes were enriched in long genes, and their association with cisplatin treatment was substantially reduced after adjustment for gene length. This finding argues against interpreting those terms as evidence of a specific neuronal transcriptional program in UT-SCC-24A cells. Rather, the length dependence provides an analytical explanation for part of the cisplatin-associated downregulation detected by RNA-seq.

The qPCR analysis performed after 72 h showed treatment-associated changes in selected apoptosis- and EMT-related transcripts. Cisplatin increased *CASP3*, *CASP9*, *BAX*, and *BCL2* mRNA levels. Under co-treatment, *BAX* mRNA expression remained similar to that observed after cisplatin alone, whereas *BCL2* mRNA expression was lower. These changes were consistent with the greater apoptotic fraction measured by Annexin V/PI staining. However, mRNA data alone cannot establish caspase activation, mitochondrial apoptosis, or a causal role for the *BAX*/*BCL2* balance. Exploratory immunoblots provided qualitative protein-level context, with a stronger BCL2 signal after cisplatin alone and a lower BCL2 signal under co-treatment, whereas BAX signal showed no clear qualitative difference among treatment conditions (Supplementary Figure S3; Supplementary Methods S2). These observations are consistent with the qPCR pattern but do not establish activation of the intrinsic apoptotic pathway. Exploratory densitometry (Supplementary Figure S4) was based on a small and variable number of valid biological replicates and showed no statistically significant differences in BCL2 or BAX relative to control after correction for multiple comparisons.

JIB-04 has been reported to modulate the activity of several JmjC-domain-containing lysine demethylases and to affect proliferation, DNA-damage responses, and drug sensitivity in cancer models [19]; however, its effects in the present models cannot be attributed to the inhibition of a specific KDM without direct target-engagement experiments. The earlier RNA-seq dataset showed expression of multiple JmjC-domain-containing KDM transcripts across the four UT-SCC cell lines, with inter-line variability. These data demonstrate basal transcript expression but do not establish KDM protein abundance, enzymatic activity, or responsiveness to JIB-04. The exploratory histone-immunoblot analysis (Supplementary Figure S3; Supplementary Methods S2) showed stronger H3K36me3 signals under co-treatment, with less consistent changes in H3K4me3, H3K9me3 and H3K27me3. In the exploratory densitometry (Supplementary Figure S4), the H3K36me3 signal under co-treatment was approximately three-fold higher than in the control; however, this difference was not statistically significant after correction for multiple comparisons. Because the H3K27me3 signal in the control was undetectable, a reliable quantitative comparison of H3K27me3 with the control was not possible. These findings are compatible with treatment-associated changes in the global histone marks examined; however, they do not identify the responsible KDMs, demonstrate direct target engagement, or establish that the changes in histone methylation mediate the response to cisplatin. The observed effects should therefore be described as pharmacological effects associated with JIB-04-containing treatment rather than as confirmed KDM-dependent epigenetic reprogramming.

The transcriptional analyses also do not establish an EMT phenotype. Cisplatin increased *VIM* and *TWIST1* transcript levels, whereas JIB-04 alone was associated with modest changes in *CDH1*, *VIM*, and *SNAI1* expression. Under co-treatment, *VIM* and *TWIST1* remained elevated. Since the analysis was limited to selected mRNA markers, the results do not demonstrate EMT induction, reversal, or maintenance at the phenotypic level. The EMT-related findings should be interpreted cautiously. RNA-seq performed after 24 h showed positive enrichment of the Hallmark EMT gene set after JIB-04 treatment and negative enrichment after cisplatin treatment, whereas the EMT gene set was not significantly enriched under co-treatment. These pathway-level results do not imply concordant regulation of every EMT-associated gene; for example, *TWIST1* and *SNAI2* were increased in the combination group at 24 h. The targeted qPCR analysis performed after 72 h showed increased *VIM* and *TWIST1* transcript levels after cisplatin exposure, with both transcripts remaining elevated under co-treatment. Differences between the RNA-seq and qPCR results may reflect the different time points, the broader pathway-level versus targeted-gene analyses, and changes in the surviving cell population after prolonged treatment. Therefore, the present data do not establish EMT induction, reversal, or a causal contribution of EMT-related transcriptional changes to the response to JIB-04 and cisplatin. Protein-level analyses and functional migration or invasion assays would be needed to determine whether treatment affects epithelial–mesenchymal plasticity.

The variability in interaction patterns across the UT-SCC panel is an important outcome of this study. Although UT-SCC-24A and UT-SCC-24B originated from a primary tumor and a lymph-node metastasis from the same patient, respectively, the localized positive interaction observed in UT-SCC-24A was not reproduced to the same extent in UT-SCC-24B or in the UT-SCC-42 pair. This heterogeneity indicates that response to JIB-04-containing treatment cannot be inferred from tumor origin alone. Potential contributors include variation in drug transport, DNA-damage response, cell-cycle control, apoptotic priming, basal transcriptional state, or chromatin-associated regulation [20]. However, these factors were not directly tested, and the present data do not identify predictive biomarkers of response. The basal KDM transcript data likewise did not reveal a simple expression pattern that accounted for the response of UT-SCC-24A.

Several limitations should be acknowledged. First, the localized positive HSA interaction was observed only in UT-SCC-24A and within a narrow concentration region. Functional, molecular, and RNA-seq analyses were therefore restricted to a single model and require confirmation in independent HNSCC cell lines, including models without a positive HSA interaction. Second, MTT assesses cellular metabolic activity and does not directly measure viable-cell number, clonogenic survival, or long-term treatment response. Third, the qPCR analysis was performed after 72 h and may partly reflect the surviving cell population. Although the 24 h RNA-seq experiment characterized earlier transcriptional responses, it was performed only in UT-SCC-24A and cannot establish causal relationships. Fourth, the study did not determine the KDM target(s) of JIB-04, demonstrate direct target engagement, or establish a causal connection between altered histone marks and response to the combination. Functional, molecular, and RNA-seq analyses were restricted to UT-SCC-24A cells, which limits the generalizability of these findings. In addition, the Western blot densitometry was exploratory: the number of technically valid biological replicates was small and differed between proteins and conditions, conditions with a single valid replicate were not analyzed statistically, and the undetectable H3K27me3 control signal precluded quantitative comparison of this mark with the control. Further validation in independent HNSCC cell lines, including models without a positive HSA interaction, is needed to determine whether the observed responses are consistent across different cellular and molecular backgrounds.

Overall, these results support further preclinical study of JIB-04–cisplatin combinations in selected HNSCC cell-line models. Future work should include systematic dose-matrix studies in additional cell lines, direct measurements of viable-cell number and clonogenic survival, comparative analyses of responsive and non-responsive models, quantitative validation of histone methylation and apoptotic markers, and direct assessment of KDM target engagement.

## 5. Conclusions

JIB-04 enhanced the response to cisplatin in a model-dependent manner in established HNSCC cell lines. A localized positive interaction was identified in UT-SCC-24A within a defined low-dose region of the tested concentration matrix, where co-treatment was associated with lower BrdU incorporation, altered cell-cycle distribution, and a higher apoptotic fraction than either single agent. Early RNA-seq analysis further showed that the combination retained JIB-04-associated integrated stress-response features together with cisplatin-associated DNA-damage and cell-cycle-related transcriptional changes.

Together, these results identify UT-SCC-24A as a model in which JIB-04 co-treatment was associated with an enhanced cellular response to cisplatin within a defined concentration range. The accompanying functional and transcriptional findings provide a framework for further investigation of the biological context associated with this response. Because the interaction was evaluated in vitro and was not uniform across the cell-line panel, its reproducibility and molecular determinants should be examined in additional HNSCC models.

## Figures and Tables

**Table 1:** Primer sequences used for RT-qPCR. Table presents the forward and reverse primer sequences used for RT-qPCR analysis of apoptosis-related genes (*CASP3*, *CASP9*, *BAX*, *BCL2*, and *TP53*), the proliferation-related gene *MYC*, epithelial–mesenchymal transition-associated genes (*VIM*, *TWIST1*, *CDH1*, and *SNAI1*) and the reference genes *GAPDH* and *ACTB*.

Gene	Forward Primer (5′-3′)	Reverse Primer (5′-3′)
*CASP3*	CATGGAAGCGAATCAATGGACT	CTGTACCAGACCGAGATGTCA
*CASP9*	CTCAGACCAGAGATTCGCAAAC	GCATTTCCCCTCAAACTCTCAA
*TP53*	CAGCACATGACGGAGGTTGT	TCATCCAAATACTCCACACGC
*MYC*	GGCTCCTGGCAAAAGGTCA	CTGCGTAGTTGTGCTGATGT
*BAX*	CCCGAGAGGTCTTTTTCCGAG	CCAGCCCATGATGGTTCTGAT
*BCL2*	GGTGGGGTCATGTGTGTGG	CGGTTCAGGTACTCAGTCATCC
*VIM*	GACGCCATCAACACCGAGTT	CTTTGTCGTTGGTTAGCTGGT
*TWIST1*	GTCCGCAGTCTTACGAGGAG	GCTTGAGGGTCTGAATCTTGCT
*CDH1*	CGAGAGCTACACGTTCACGG	GGGTGTCGAGGGAAAAATAGG
*SNAI1*	TCGGAAGCCTAACTACAGCGA	AGATGAGCATTGGCAGCGAG
*GAPDH*	GGAGCGAGATCCCTCCAAAAT	GGCTGTTGTCATACTTCTCATGG
*ACTB*	CATGTACGTTGCTATCCAGGC	CTCCTTAATGTCACGCACGAT

## Data Availability

Data is contained within the article or Supplementary Materials.

## Supplementary Materials

The following supporting information can be downloaded at https://www.mdpi.com/article/10.3390/cells15212023/s1: Figure S1: Basal expression of selected JmjC-domain-containing lysine demethylase genes across four UT-SCC cell lines; Figure S2: Principal component analysis of RNA-seq profiles from UT-SCC-24A cells following treatment with JIB-04, cisplatin, or their combination; Figure S3: Exploratory Western blot analysis of selected histone methylation marks, BAX, and BCL2 in UT-SCC-24A cells; Figure S4: Densitometric quantification of the Western blot analysis shown in Figure S3; Table S1: Publicly available provenance and selected molecular annotations of the UT-SCC cell lines used in this study; Supplementary Methods S1: RNA-seq processing and bioinformatic analysis; Supplementary Methods S2: Exploratory Western blot analysis of histone methylation marks, BAX, and BCL2.

## Abbreviations

The following abbreviations are used in this manuscript:

ACTB	Actin beta
BAX	BCL2-associated X protein
BCL2	B-cell lymphoma 2
BrdU	5-Bromo-2′-deoxyuridine
cDNA	Complementary DNA
CTNNB1	Catenin beta 1
DAPI	4′,6-Diamidino-2-phenylindole
DMEM/F12	Dulbecco’s Modified Eagle Medium/Nutrient Mixture F-12
DMSO	Dimethyl sulfoxide
EMT	Epithelial–mesenchymal transition
FBS	Fetal bovine serum
GAPDH	Glyceraldehyde-3-phosphate dehydrogenase
HNSCC	Head and neck squamous cell carcinoma
HSA	Highest Single Agent
IC_50_	Half-maximal inhibitory concentration
JIB-04	Jumonji domain-containing histone demethylase inhibitor 04
JmjC	Jumonji C domain
KDM	Histone lysine demethylase
KMT	Histone lysine methyltransferase
LDH	Lactate dehydrogenase
LSD	Lysine-specific demethylase
MTT	3-(4,5-Dimethylthiazol-2-yl)-2,5-diphenyltetrazolium bromide
PI	Propidium iodide
qPCR	Quantitative polymerase chain reaction
RQ	Relative quantification
UT-SCC	University of Turku squamous cell carcinoma
WNT	Wingless-related integration site
