# Supplementary material for: Enhancement of Cisplatin Efficacy by the Histone Demethylase Inhibitor JIB-04 in Head and Neck Squamous Cell Carcinoma (HNSCC) Cell Lines
Source: Cells. 2026 Nov 6;15(21):2023. doi: 10.3390/cells15212023 (PMC13761571; doi:10.3390/cells15212023)
Supplement: Supplementary file 1 [file cells-15-02023-s001.zip › Supplementary Figures.pdf]

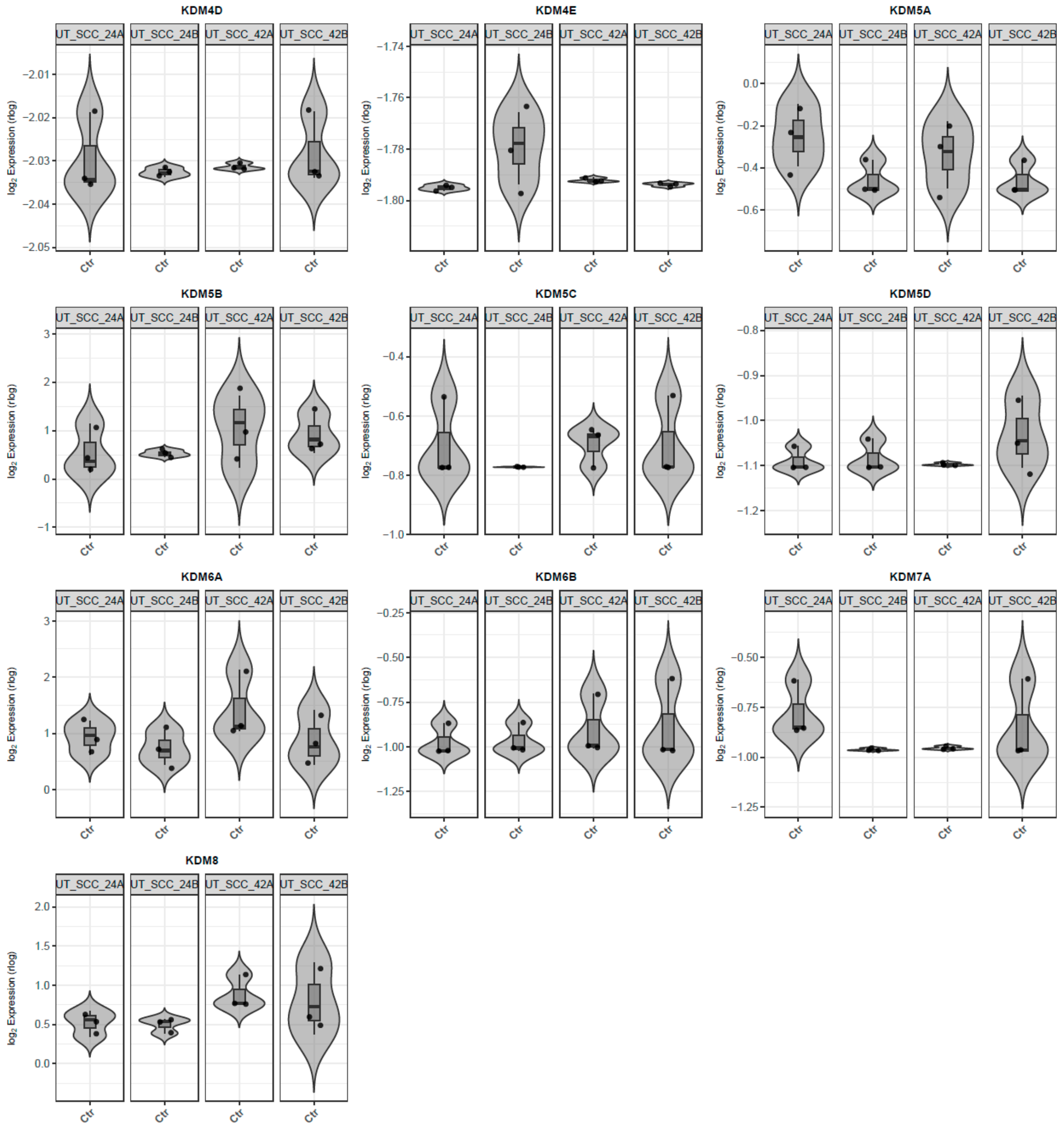

Figure S1. Basal expression of selected histone lysine demethylase genes in established HNSCC cell lines. Violin plots show regularised-logarithm (rlog)-transformed RNA-seq expression values for KDM4D, KDM4E, KDM5A, KDM5B, KDM5C, KDM5D, KDM6A, KDM6B, KDM7A, and KDM8 in untreated UT-SCC-24A, UT-SCC-24B, UT-SCC-42A, and UT-SCC-42B cells. Points represent individual biological replicates, and embedded box plots summarise the distribution within each group. These data were obtained from a separate baseline RNA-seq dataset and are presented descriptively to illustrate basal transcript expression across the cell-line panel; they do not establish differences in KDM protein abundance or enzymatic activity.

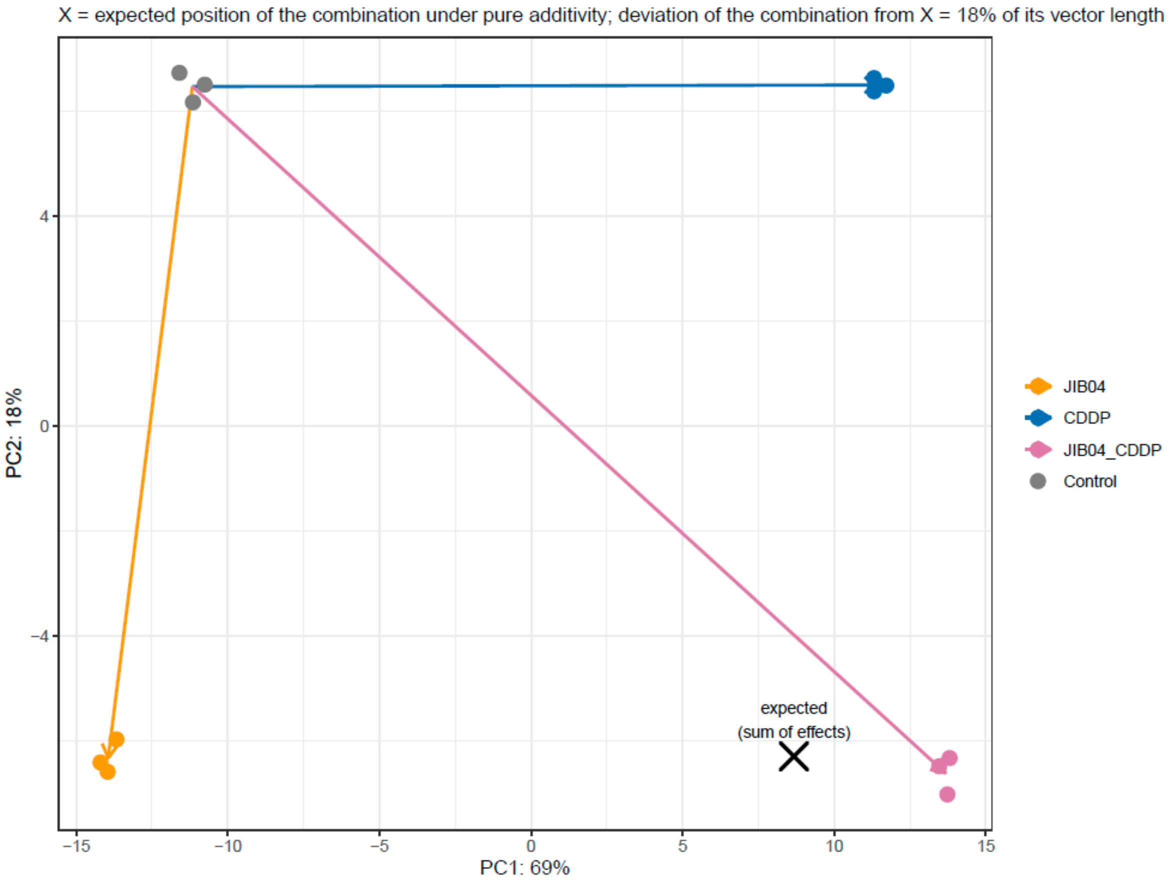

Figure S2. Principal component analysis of transcriptional responses to JIB-04, cisplatin, and their combination in UT-SCC-24A cells. Cells were treated for 24 h with vehicle control, 0.075 µM JIB-04, 6.935 µM cisplatin, or their combination. PCA was performed on variance-stabilised expression data for the 500 most variable genes. Points represent three independent biological replicates per treatment group. PC1 and PC2 explain 69% and 18% of the variance, respectively. Arrows indicate treatment-associated displacements from the control centroid to the corresponding treatment centroids. The black cross marks the expected additive combination position in the PC1–PC2 plane, calculated as centroid(JIB-04) + centroid(cisplatin) − centroid(control). The distance between the observed combination centroid and this expected position corresponds to 18% of the control-to-observed-combination distance. This percentage is a descriptive geometric measure, unrelated to the 18% of variance explained by PC2, and was not used for statistical inference or interpreted as a measure of pharmacological synergy.

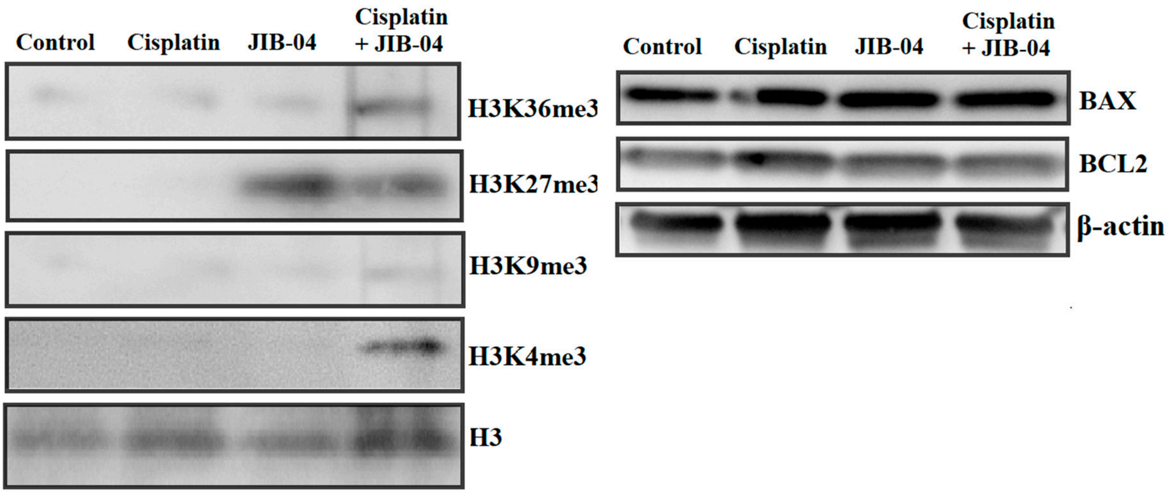

Figure S3. Exploratory Western blot analysis of histone methylation marks and apoptosis-related proteins in UT-SCC-24A cells. Cells were treated for 48 h with vehicle control, 6.935 µM cisplatin, 0.075 µM JIB-04, or their combination. Histone extracts were analysed for H3K36me3, H3K27me3, H3K9me3, and H3K4me3, with total histone H3 serving as the loading reference. BAX and BCL2 were analysed in whole-cell lysates, with β-actin serving as the loading reference. The blots shown are representative of three independent biological experiments.

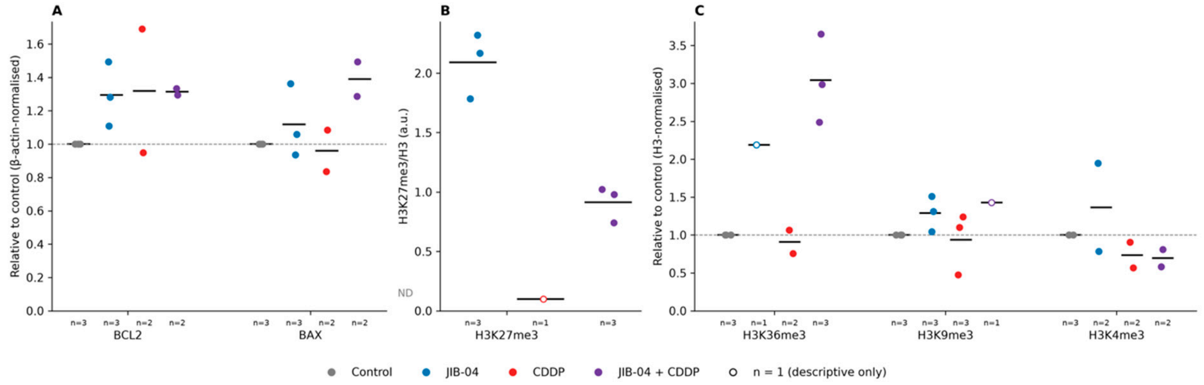

Figure S4. Exploratory densitometric quantification of the Western blot analysis shown in Supplementary Figure S3. Cells were treated for 48 h with vehicle control, 0.075 µM JIB-04, 6.935 µM cisplatin (CDDP), or their combination. (A) BCL2 and BAX, normalised to β-actin. (B) H3K27me3, normalised to total histone H3. (C) H3K36me3, H3K9me3 and H3K4me3, normalised to total histone H3. Each dot represents an independent biological replicate; the horizontal line indicates the mean. The experiments were performed in three independent biological experiments; part of the densitometric measurements did not meet the technical quality criteria, and the number of valid biological replicates therefore differs between proteins and conditions (n is shown below each group). Numbers of valid biological replicates (control/JIB-04/CDDP/combination): BCL2 3/3/2/2; BAX 3/3/2/2; H3K36me3 3/1/2/3; H3K9me3 3/3/3/1; H3K4me3 3/2/2/2; H3K27me3 ND/3/1/3. In (A) and (C), values are expressed relative to the control of the same experiment (set to 1.0, dashed line). Treatment effects were tested on log2-transformed treatment/control ratios by two-sided one-

sample t-tests, with Holm correction across all 13 evaluable comparisons; no comparison was statistically significant after correction (all adjusted $p \geq 0.13$). Conditions with a single valid biological replicate (open symbols) are shown for descriptive purposes only and were not analysed statistically. In (B), the control signal was below the detection limit and is shown as not detected (ND); it was not assigned a value of zero. H3K27me3 values are shown as H3K27me3/H3 ratios in arbitrary units, no fold change relative to control was calculated, and no statistical testing was performed.
