# Supplementary material for: Enhancement of Cisplatin Efficacy by the Histone Demethylase Inhibitor JIB-04 in Head and Neck Squamous Cell Carcinoma (HNSCC) Cell Lines
Source: Cells. 2026 Nov 6;15(21):2023. doi: 10.3390/cells15212023 (PMC13761571; doi:10.3390/cells15212023)
Supplement: Supplementary file 1 [file cells-15-02023-s001.zip › Supplementary Methods.pdf]

Supplementary Methods S1. RNA-seq analysis of UT-SCC-24A cells treated with JIB-04, cisplatin and their combination

Experimental groups

The analysis comprised 12 RNA samples of the UT-SCC-24A cell line in four groups (n = 3 biological replicates each): untreated control (Control), JIB-04 (JIB04), cisplatin (CDDP) and the combination of JIB-04 and cisplatin (JIB04+CDDP). Sample annotation (sample ID, cell line, treatment, replicate) was taken from the RNA quantification sheet provided with the samples.

Read processing and quantification

Paired FASTQ files from two NovaSeq X flow cells were processed with the nf-core/rnaseq pipeline (version 3.13.2; Nextflow, Docker profile). Reads of the same sample sequenced on the two flow cells were merged by the pipeline before analysis. Adapter and quality trimming was performed with Trim Galore, reads were aligned to the human reference genome GRCh38 (Ensembl release 110, soft-masked primary assembly, Ensembl 110 gene annotation) with STAR, and gene-level expression was quantified with Salmon in alignment-based mode (pipeline default "star_salmon" route). Library strandedness was inferred automatically by the pipeline. Gene-level counts scaled to library size and average transcript length ("length-scaled" counts produced by tximport within the pipeline) were rounded to integers and used for all downstream analyses. Quality metrics reported per sample were taken from the STAR alignment logs (number of input reads, percentage of uniquely mapped reads, percentage of reads mapped to multiple loci, percentage of reads unmapped because too short) and from the Salmon count matrix (total number of reads assigned to genes and number of genes with at least 10 assigned reads).

Gene annotation and filtering

Gene biotype and gene length were taken from the Ensembl 110 GTF file. Gene length was defined as the genomic span of the gene, i.e. the distance from the first to the last base of the gene locus (end - start + 1), including introns. This measure, rather than the length of the mature transcript, was used because it reflects the distance that RNA polymerase II must travel to transcribe the gene, which is the relevant quantity when transcription is impeded by DNA lesions (see "Gene-length analysis").

Genes annotated as ribosomal RNA (biotypes rRNA, rRNA_pseudogene and Mt_rRNA) were removed, because multi-copy rRNA genes (e.g. 5.8S rRNA paralogs) receive reads that are distributed arbitrarily between identical copies, which produces spurious "all-or-nothing" count patterns and artificially extreme fold changes. In addition, a gene was retained only if it had at least 10 reads in all three replicates of at least one experimental group. This filter keeps genes that are reliably expressed in at least one condition (including genes switched on or off by a treatment) while removing genes whose counts are driven by single samples. After filtering, 15,329 genes were analysed.

Differential expression analysis

Differential expression was analysed with DESeq2 (version 1.46.0) using a model with one factor of four levels (design ~ treatment). DESeq2 normalises counts for differences in sequencing depth between samples (median-of-ratios method) and tests each gene with a Wald test. Six comparisons (contrasts) were evaluated: JIB04 vs Control, CDDP vs Control, JIB04+CDDP vs Control, JIB04+CDDP vs JIB04, JIB04+CDDP vs CDDP, and the drug interaction (defined below).

Interpretation of the reported metrics:
- log2 fold change (log2FC) is the base-2 logarithm of the ratio of normalised expression between two groups; log2FC = 1 corresponds to a two-fold increase, log2FC = -1 to a two-fold decrease and log2FC = 0 to no change.
- Shrunken log2FC (log2FC_shrunk) is the log2FC after adaptive shrinkage with the ashr method (ashr version 2.2-63). Shrinkage pulls imprecise estimates (typically from lowly expressed genes with high variance) towards zero, while precise estimates remain essentially unchanged. Shrunken values were used to rank genes and to display effect sizes; statistical significance and the fold-change threshold were always based on the unshrunken estimates and their p-values.
- padj is the p-value adjusted for multiple testing with the Benjamini-Hochberg procedure (false discovery rate). DESeq2 additionally applies independent filtering: genes whose mean expression is too low to reach significance in a given comparison are excluded from the multiple-testing correction for that comparison and receive padj = NA ("not tested"). Because this filter is optimised separately for each comparison, the number of tested genes differs between comparisons; it was lowest for the interaction contrast (11,463 of 15,329 genes), which has the lowest statistical power.
- A gene was considered differentially expressed (DE) when padj < 0.05 and |log2FC| > 1 (at least two-fold change). The number of genes with padj < 0.05 irrespective of fold change is also reported.

Drug interaction (deviation from additivity)
The interaction contrast measures whether the combination behaves differently from what is expected if the two drugs acted independently. It was estimated within the same DESeq2 model as

interaction = log2FC(JIB04+CDDP vs Control) - log2FC(JIB04 vs Control) - log2FC(CDDP vs Control).

Because log2FC values are on a logarithmic scale, independent (additive on the log scale, i.e. multiplicative on the linear scale) action of the two drugs gives an interaction of zero. A positive interaction means that the gene is expressed higher in the combination than expected from the sum of the single-drug effects, a negative interaction that it is expressed lower than expected. The biological meaning depends on the direction of the combination effect: an interaction with the same sign as the combination effect indicates a stronger-than-additive response, whereas an interaction with the opposite sign indicates a weaker-than-additive response. The interaction must therefore always be interpreted together with the single-drug and combination effects.

Additivity classes
For every gene that was DE in at least one treatment versus Control (JIB04, CDDP or JIB04+CDDP; n = 1,682), the expected effect of the combination under additivity was calculated as log2FC(JIB04) + log2FC(CDDP) and compared with the observed log2FC of the combination (unshrunken values, i.e. the same scale as the interaction test). Genes were classified as: "no statistically significant interaction detected" (interaction padj >= 0.05; a non-significant interaction test does not establish additivity); "stronger than sum" (interaction padj < 0.05, observed and expected effects in the same direction and |observed| > |expected|); "weaker than sum" (interaction padj < 0.05, same direction, |observed| < |expected|); "opposite direction to sum" (interaction padj < 0.05, observed and expected effects of opposite sign); and "interaction not tested" (interaction padj = NA because of independent filtering, see above).

Global structure of the data

Counts were transformed with the variance-stabilising transformation (VST, DESeq2, blind to the experimental design), which puts expression on an approximately log2 scale with comparable variance across the expression range. Principal component analysis (PCA) was performed on the 500 genes with the highest variance across samples. To visualise how the combination relates to the single drugs, the centroid (mean position) of each group was calculated and the drug effects were represented as vectors from the Control centroid to each treatment centroid. The position expected for the combination under additivity was calculated as centroid(JIB04) + centroid(CDDP) - centroid(Control). The deviation of the combination was expressed as the distance between the observed and the expected combination centroids divided by the length of the observed combination vector (Control to JIB04+CDDP); 0% indicates perfect additivity. Sample-to-sample similarity was assessed by Euclidean distances between VST profiles.

Overlap of DE genes and expression-pattern clusters

DE genes of the three treatment-versus-Control comparisons were assigned to categories according to the comparisons in which they were significant (e.g. "Combo" = significant only in the combination; "CDDP + Combo" = significant after CDDP alone and in the combination), separately for up- and down-regulated genes. For clustering, the VST expression of all DE genes (n = 1,682) was converted to z-scores per gene (the mean of each gene across the 12 samples set to 0 and its standard deviation to 1), so that clustering reflects the pattern of change rather than expression level. Genes were clustered hierarchically (Euclidean distance, Ward.D2 linkage) and the tree was cut into six clusters; the pattern of each cluster is summarised by the mean z-score of its genes in each group.

Functional enrichment

Over-representation analysis (ORA) of Gene Ontology Biological Process (GO BP) terms was performed for each cluster with clusterProfiler (version 4.14.6; org.Hs.eg.db 3.20.0, GO.db 3.20.0). ORA tests whether a GO term contains more genes from a given gene list than expected by chance; all 15,329 filtered genes were used as the background. Terms with Benjamini-Hochberg padj < 0.05 were considered significant.

Gene set enrichment analysis (GSEA) was performed for four comparisons (JIB04 vs Control, CDDP vs Control, JIB04+CDDP vs Control and the interaction) using the MSigDB Hallmark collection (50 gene sets representing well-defined biological states and processes; obtained with msigdbr 25.1.1) and GO BP terms (gene set size 15-500 genes). For GSEA, all genes with a test statistic were ranked by the DESeq2 Wald statistic (log2FC divided by its standard error; positive for up-regulated and negative for down-regulated genes), so that both the direction and the reliability of each change contribute to the ranking; no significance threshold is applied to individual genes. GSEA was run with the fgsea algorithm (fgsea 1.32.4) as implemented in clusterProfiler, and p-values were adjusted with the Benjamini-Hochberg method (padj < 0.05 considered significant). The normalised enrichment score (NES) summarises whether genes of a set are concentrated at the top (NES > 0, the pathway is activated/up-regulated) or at the bottom (NES < 0, the pathway is suppressed/down-regulated) of the ranking; the absolute value reflects the strength of the enrichment corrected for gene set size. For the interaction contrast, NES > 0 means that the pathway is higher in the combination than expected from the sum of the single-drug effects and NES < 0 that it is lower than expected (see "Drug interaction"). Because

NES values are not additive quantities, pathway-level interactions were interpreted qualitatively together with the NES of the single drugs and of the combination. For gene symbols represented by more than one Ensembl gene, the gene with the largest absolute statistic was used.

Key genes in biological modules

To relate the global results to specific processes, expression changes of selected marker genes were examined in eight predefined modules: integrated stress response/ATF4 targets, p53/DNA damage response, proliferation, Notch signalling, hypoxia/HIF targets, inflammation/NF-kB, epithelial-mesenchymal transition (EMT) and JmjC-domain lysine demethylases (KDM). Shrunken log2FC values are shown for the treatment-versus-Control comparisons and unshrunken values for the interaction.

Gene-length analysis

Transcription-blocking DNA lesions such as cisplatin adducts stall RNA polymerase II; the probability that a gene is transcribed to completion therefore decreases with gene length, and long genes are expected to be repressed preferentially. To test for this effect, genes were divided into ten equally sized groups (deciles) according to gene length (log10 genomic span), and the median shrunken log2FC was calculated in each decile for each comparison. A decrease of the median log2FC from short to long genes indicates length-dependent repression. The association between log2FC and gene length was quantified with the Spearman rank correlation coefficient (rho; negative values mean that longer genes are more strongly down-regulated). To test whether the enrichment of neuronal/synaptic GO terms among cisplatin-repressed genes reflects gene length rather than a specific biological programme, genes annotated to GO:0050808 "synapse organization" (including all child terms) were compared with all other genes: their effect in CDDP vs Control was estimated by linear regression of the shrunken log2FC on term membership, without and with adjustment for gene length (log10 length and length decile). A strong reduction of the effect after adjustment indicates that the signal is largely explained by gene length. In addition, every GO term in the enrichment tables was annotated with the median length of its genes, and terms whose genes had a median length more than twice the median length of all analysed genes (30.2 kb) were flagged as "long-gene terms", i.e. terms whose enrichment may be driven by gene length.

Software

Statistical analyses were performed in R (version 4.4.1) with DESeq2 1.46.0, ashr 2.2-63, clusterProfiler 4.14.6, enrichplot 1.26.6, DOSE 4.0.1, fgsea 1.32.4, org.Hs.eg.db 3.20.0, GO.db 3.20.0, msigdbr 25.1.1, pheatmap 1.0.13 and ggplot2 4.0.3. Read processing used the software versions bundled with nf-core/rnaseq 3.13.2.

Supplementary Methods S2. Exploratory Western blot analysis of histone methylation marks, BAX, and BCL2

UT-SCC-24A cells were seeded at a density of $5x10^4$ cells/ml in 6-well plates. On the following day, cells were treated with DMSO, 0.075 μM JIB-04, 6.935 μM cisplatin, or their combination for 48 hours.

The 48-hour time point was selected primarily to assess treatment-associated changes in histone methylation after sustained drug exposure, while limiting the potential contribution of

downstream effects associated with the longer, 72-hour treatment period used in the functional assays. BAX and BCL2 were assessed at the same time point to provide complementary information on apoptosis-related protein expression under the same treatment conditions. This time point differs from those used for RNA-seq (24 h) and the qPCR and functional assays (72 h); the Western blot findings were therefore interpreted as protein-level observations under matched treatment conditions rather than as direct time-matched validation of the transcriptional or functional results.

For the analysis of histone methylation, cells were harvested, washed twice with ice-cold PBS, and resuspended in Triton extraction buffer (TEB; 0.5% Triton X-100, 2 mM PMSF, and 0.02% NaN_3 in PBS, pH 7.4). Lysates were centrifuged at 800 × g for 10 min at 4 °C. The nuclear pellet was washed once with TEB and centrifuged again under the same conditions. Histones were extracted from the resulting pellet with 0.02 N HCl overnight at 4 °C. Samples were then centrifuged at 12,000 × g for 10 min at 4 °C, and the histone-containing supernatant was collected for Western blot analysis. Histone concentration was determined using Pierce™ 660nm Protein Assay Kit (catalogue no. 22662; Thermo Scientific). Equal amounts of histones (30 ug) were mixed with 4× Laemmli sample buffer and heated at 95 °C for 5 min. Samples were separated using 4–20% Mini-PROTEAN TGX Stain-Free gels (catalogue no. 4568095; Bio-Rad) and transferred onto PVDF membranes. After blocking with EveryBlot Blocking Buffer (catalogue no. 12010020; Bio-Rad), membranes were incubated overnight at 4 °C with primary antibodies against H3K4me3 (MA5-11199), H3K9me3 (710816), H3K27me3 (PA5-31817), or H3K36me3 (MA5-24687), as appropriate. Total histone H3 was detected using antibody PA5-16183.

For the analysis of BAX and BCL2, whole-cell proteins were extracted separately using RIPA Lysis and Extraction Buffer (catalogue no. 89901; Thermo Scientific) supplemented with Halt™ Protease and Phosphatase Inhibitor Cocktail (100X) (catalogue no. 78440; Thermo Scientific). Protein concentrations were determined using Pierce™ 660nm Protein Assay Kit (catalogue no. 22662; Thermo Scientific). Equal amounts of protein (30 ug) were separated using 4–20% Mini-PROTEAN TGX Stain-Free gels (catalogue no. 4568095; Bio-Rad) and transferred onto PVDF membranes. After blocking with EveryBlot Blocking Buffer (catalogue no. 12010020; Bio-Rad), membranes were incubated overnight at 4 °C with primary antibodies against BAX (50599-2-Ig), BCL2 (PA5-27094) and β-Actin (MA5-33078) which served as the loading reference for proteins analyzed in RIPA lysates.

The HRP-conjugated goat anti-rabbit IgG (H+L) secondary antibody (catalogue no. 31460) was used to detect BAX, BCL2, and the histone methylation marks, as all corresponding primary antibodies were raised in rabbit. After incubation with secondary antibody, protein bands were detected using Clarity Max Western ECL Substrate (catalogue no. 1705062; Bio-Rad) and imaged using a G:BOX Chemi XR5 imaging system (Syngene, Cambridge, UK).

If required, membranes were stripped using Restore Western Blot Stripping Buffer (catalogue no. 21059; Thermo Scientific), according to the manufacturer's instructions.

Band intensities were quantified using ImageJ software (version 1.54t; National Institutes of Health, Bethesda, MD, USA). Histone methylation signals were normalised to total histone H3, and BAX and BCL2 signals were normalised to β-actin. Normalised values were expressed

relative to the corresponding vehicle control within each independent biological experiment. For H3K27me3, the signal in the corresponding vehicle control was below the detection limit; the control signal was reported as not detected (ND) rather than as zero, and H3K27me3 values are therefore reported as H3K27me3/H3 ratios in arbitrary units rather than as fold-changes relative to control.

The experiments were performed in three independent biological experiments. Because part of the densitometric measurements did not meet the technical quality criteria, the number of technically valid measurements differs between proteins and conditions; the exact number of independent biological replicates for each protein and condition is given in the legend of Supplementary Figure S4. Conditions with a single valid biological replicate (H3K36me3 after JIB-04, H3K9me3 after JIB-04 + cisplatin and H3K27me3 after cisplatin) are shown for descriptive purposes only and were excluded from all statistical tests. Because each value was normalised to the vehicle control of the same independent experiment (control = 1.0), treatment effects for BCL2, BAX, H3K36me3, H3K9me3 and H3K4me3 were tested on log2-transformed treatment/control ratios using a two-sided one-sample t-test against 0 (corresponding to a geometric-mean ratio of 1). P-values were adjusted for multiple comparisons using the Holm method across all 13 evaluable comparisons ($n = 2–3$ per comparison); adjusted $p < 0.05$ was considered statistically significant. For H3K27me3, the signal in the vehicle control was below the detection limit. Undetectable control signals were reported as not detected (ND) and were not assigned a value of zero; H3K27me3 values are therefore reported as H3K27me3/H3 ratios in arbitrary units, no fold change relative to control was calculated, and no statistical testing was performed for this mark. Given the small number of replicates, all densitometric analyses are considered exploratory. Statistical analyses were performed in Python (SciPy, version 1.18.1).
