# Supplementary material for: Enhancement of Cisplatin Efficacy by the Histone Demethylase Inhibitor JIB-04 in Head and Neck Squamous Cell Carcinoma (HNSCC) Cell Lines
Source: Cells. 2026 Nov 6;15(21):2023. doi: 10.3390/cells15212023 (PMC13761571; doi:10.3390/cells15212023)
Supplement: Supplementary file 1 [file cells-15-02023-s001.zip › Supplementary Table.pdf]

Supplementary Table S1. Publicly available provenance and selected molecular annotations of the UT-SCC cell lines used in this study.

Cell line	RRID / Cellosaurus accession	Tumor type and anatomical site	Source material	Donor sex / age	Matched line	HPV status	TP53 status reported in Cellosaurus
UT-SCC-24A	RRID:CVCL_7826	Tongue squamous cell carcinoma; oral cavity/tongue	Primary tumor	Male / 41 years	UT-SCC-24B	HPV-negative	Homozygous splice-acceptor variant: *TP53* c.673-2A>T (IVS6-2A>T); ClinVar VCV000635388
UT-SCC-24B	RRID:CVCL_7827	Tongue squamous cell carcinoma; cervical lymph-node metastasis	Cervical lymph-node metastasis	Male / 41 years	UT-SCC-24A	HPV-negative	Splice-acceptor variant: *TP53* c.673-2A>T (IVS6-2A>T); zygosity not specified
UT-SCC-42A	RRID:CVCL_7847	Laryngeal squamous cell carcinoma; supraglottic larynx	Primary tumor	Male / 43 years	UT-SCC-42B	HPV-negative	Homozygous nonsense variant: *TP53* p.Gln331Ter (c.991C>T); ClinVar VCV000922893
UT-SCC-42B	RRID:CVCL_7848	Laryngeal squamous cell carcinoma; cervical lymph-node metastasis	Cervical lymph-node metastasis	Male / 43 years	UT-SCC-42A	HPV-negative	No specific *TP53* sequence variant is displayed in the Cellosaurus entry
